# Supplementary material for: Protocol for a randomised controlled trial to evaluate the effectiveness of the ‘Care for Stroke’ intervention in India: a smartphone-enabled, carer-supported, educational intervention for management of disabilities following stroke
Source: BMJ Open. 2018 May 9;8(5):e020098. doi: 10.1136/bmjopen-2017-020098 (PMC5942432; doi:10.1136/bmjopen-2017-020098)
Supplement: Author's manuscript [file bmjopen-2017-020098.draft_revisions.pdf]

BMJ Open is committed to open peer review. As part of this commitment we make the peer review history of every article we publish publicly available.

When an article is published we post the peer reviewers' comments and the authors' responses online. We also post the versions of the paper that were used during peer review. These are the versions that the peer review comments apply to.

The versions of the paper that follow are the versions that were submitted during the peer review process. They are not the versions of record or the final published versions. They should not be cited or distributed as the published version of this manuscript.

BMJ Open is an open access journal and the full, final, typeset and author-corrected version of record of the manuscript is available on our site with no access controls, subscription charges or pay-per-view fees (http://bmjopen.bmj.com).

If you have any questions on BMJ Open's open peer review process please email info.bmjopen@bmj.com

BMJ Open

Protocol for a randomised controlled trial to evaluate the effectiveness of the 'care for stroke' intervention in India; a smartphone-enabled, carer-supported, educational intervention for management of disabilities following stroke

Journal:	*BMJ Open*
Manuscript ID	bmjopen-2017-020098
Article Type:	Protocol
Date Submitted by the Author:	13-Oct-2017
Complete List of Authors:	Sureshkumar, K; Public Health Foundation of India, SACDIR; London School of Hygiene and Tropical Medicine, International Center for Evidence in Disability<br>Murthy, GVS; MBBS, MD (Community Medicine) and MSc (Community Eye Health), Director, IIPH Hyderabad.<br>Kuper, Hannah; The London School of Hygiene & Tropical Medicine, Clinical Research
Keywords:	Clinical trials < THERAPEUTICS, Stroke < NEUROLOGY, Disability, mHealth, REHABILITATION MEDICINE

SCHOLARONE™
Manuscripts

For peer review only

Protocol for a randomised controlled trial to evaluate the effectiveness of the 'care for stroke' intervention in India; a smartphone-enabled, carer-supported, educational intervention for management of disabilities following stroke

K Sureshkumar[1], GVS Murthy[2], Hannah Kuper[3]

Corresponding Author:

Sureshkumar Kamalakannan

Indian Institute of Public Health - Hyderabad

Plot No: 1 ANV Arcade, Amar Cooperative Society

Kavuri Hills, Madhapur Hyderabad - 500081

Email: suresh.kumar@iiphh.org

Phone: +91 9676333412, +91 9840772381.

Authors

1. Public Health Foundation of India, Indian institute of Public Health - Hyderabad, Plot 1 ANV Arcade, Amar Cooperative Society, Kavuri Hills, Madhapur, Hyderabad, Telangana, 500081. suresh.kumar@iiphh.org
2. Public Health Foundation of India, Indian institute of Public Health - Hyderabad, Plot 1 ANV Arcade, Amar Cooperative Society, Kavuri Hills, Madhapur, Hyderabad, Telangana, 500081. murthy.gvs@iiphh.org
3. International centre for Evidence in Disability, Department of Clinical Research, London School of Hygiene and Tropical Medicine, Keppel Street, London, WC1E 7HT. Hannah.Kuper@lshtm.ac.uk

For peer review only - http://bmjopen.bmj.com/site/about/guidelines.xhtml

Keywords

1. Clinical Trial
2. Stroke
3. Disability
4. Mhealth
5. Rehabilitation
6. Clinical effectiveness

Word Count: Manuscript: - 2613

For peer review only

For peer review only - http://bmjopen.bmj.com/site/about/guidelines.xhtml

Abstract:

Introduction: The increase in prevalence of stroke and stroke-related disability implies an overwhelming demand for rehabilitation services worldwide. This situation is especially true for country like India where the resources for rehabilitation are very limited. Recently, a smartphone-enabled carer-supported educational intervention for management of physical disabilities following stroke was developed in India. It was found feasible and acceptable in an Indian context. The intervention now needs to be evaluated for its clinical effectiveness through a randomized controlled trial.

Methods: This trial will be a multi-center, pragmatic, randomised, outcome assessor-blind, controlled trial to quantify the effectiveness of the Care for Stroke Intervention on reducing dependency in activities of daily living following stroke. A total of 320 adult stroke survivors who fulfil the eligibility criteria will be randomised to receive either 'Care for Stroke' intervention or standard treatment and will be followed up for six weeks.

Analysis: The main analyses will compare all those participants allocated to the 'Care for Stroke' intervention versus those allocated to the standard treatment group on an 'intention-to-treat' basis, irrespective of whether the participants received the

For peer review only

For peer review only - http://bmjopen.bmj.com/site/about/guidelines.xhtml

treatment allocated or not. Appropriate effect estimates with a measure of precision (95% confidence interval) will be presented in results of the trial.

Ethics and Dissemination: The Indian Institute of Public Health-Hyderabad / Public Health Foundation of India – Independent Institutional Ethics Committee; Peer reviewed Publications.

Registration Details: Clinical Trial Registry of India CTRI/2017/07/009014.

For peer review only

For peer review only - http://bmjopen.bmj.com/site/about/guidelines.xhtml

Strengths and Limitations of the study:

1. The trial protocol is rigorously designed and hence the results are expected to be accurate.
2. The methods were pilot-tested previously and hence conduct of the trial will be highly scientific and feasible.
3. The funding is limited to an Early Career Fellowship hence much of the work will have to efficiently planned and implemented.
4. The intervention is complex and hence it will be challenging to identify the exact component that may influence the effectiveness.
5. The awareness about stroke rehabilitation is very poor in the context and hence recruitment of participants will be time consuming.

For peer review only - http://bmjopen.bmj.com/site/about/guidelines.xhtml

Introduction

Globally around 15 million people suffer from stroke each year and a quarter of them experience permanent disability[1]. Much of this burden is borne by Low and Middle Income Countries (LMICs) [2]. The increase in prevalence of stroke and stroke-related disability implies an overwhelming demand for rehabilitation services worldwide[3]. This situation is especially true for LMICs like India where the resources for rehabilitation are very limited[3].

Stroke is one of the leading causes of death and disability in India. Given the paucity of data on stroke in India, a systematic review of population-based studies on stroke in India was conducted. Studies included in this review showed that the crude stroke prevalence during the past two decades in India ranged from 44.29/100,000 persons to 559/100,000 persons in different parts of the country[4]. During the past two decades, the cumulative incidence of stroke in India varied widely, from 105-152/100,000 person per year in different parts of the country[4]. These estimates on stroke incidence and prevalence are found to be higher than those reported from High Income Countries[5.] The growing burden of stroke-related disability and the unmet need for rehabilitation following stroke in India poses a major public health challenge.

For peer review only - http://bmjopen.bmj.com/site/about/guidelines.xhtml

For peer review only

Given this challenge, it is imperative to develop cost-effective multi-dimensional stroke rehabilitation interventions to meet the demands of the stroke survivors. In the absence of any organised stroke care services, and with the limited resources available for rehabilitation, a comprehensive approach to address the growing burden of stroke-related disability in India becomes pertinent[6]. This approach could be pivotal in integrating various strategies for rehabilitation[3] (Educational, Community-based rehabilitation, digital technology, Self/Supported management etc.). It could also be useful for targeting the full range of impacts of stroke, including on impairments, activity limitations and participation restriction, as outlined in the 'Biopsychosocial conceptualization of disability framework' for the intervention, as proposed by the ICF[7].

As a part of the author's doctoral study, a smartphone-enabled carer-supported educational intervention was developed for the management of physical disabilities following stroke in India[8]. This intervention was named as 'Care for Stroke'. It was developed using the systematic approach to development and evaluation of complex interventions, as recommended by the Medical Research Council (MRC) in the U.K. [9-10]. To the best of our knowledge, there is no other stroke rehabilitation intervention enabled through mHealth platforms that are available and relevant to India.

For peer review only - http://bmjopen.bmj.com/site/about/guidelines.xhtml

Following development, the intervention was evaluated for its feasibility and acceptability in an Indian context[11]. The intervention includes information about stroke and the ways to manage physical disability following stroke. It contains a practical demonstration of functional post-stroke exercises to acquire the functional abilities necessary to perform everyday tasks, adaptive techniques to perform one's own daily activities independently and a specific section on assistive devices that could enable participation of the stroke survivors in their daily tasks[8]. Findings from the pilot-testing showed that the 'Care for Stroke' intervention was feasible and acceptable in the Indian context[11]. About 95% of the stroke survivors and all the caregivers (100%) rated the intervention as "excellent", based on it's a) overall credibility, b) feasibility and c) user-friendliness[11].

However, feasibility and acceptability alone will not be sufficient to inform implementation and scalability[10]. Neither will it be enough in order to advocate for change in policy towards implementation of an intervention[12]. Therefore, as a next step and as recommended by the MRC, the 'Care for Stroke' intervention needs to be evaluated for its clinical and cost effectiveness in an Indian context through a randomised controlled trial.

For peer review only - http://bmjopen.bmj.com/site/about/guidelines.xhtml

Objective:

To assess whether the 'Care for Stroke' intervention is effective for the reduction of dependency in activities of daily living among stroke survivors

Methods:

Overview

This trial will be a pragmatic, randomised, outcome assessor-blinded trial to quantify the effectiveness of the Care for Stroke Intervention on reducing dependency in activities of daily living following stroke. A total of 320 adult stroke survivors who fulfil the eligibility criteria will be randomised to receive either 'Care for Stroke' intervention or standard treatment and will be followed for six weeks. The eligibility criteria will be based on uncertainty principle.

Pragmatic design and the uncertainty principle

The effectiveness of the intervention in actual everyday routine practice can be assessed using the pragmatic trial design. Until now, there is no evidence for effectiveness of stroke rehabilitation interventions that is unidisciplinary, led by a physician, neurologist or a physiotherapist alone. However, a physiotherapist or physician-driven unidisciplinary rehabilitation is what is commonly practiced in the context of stroke rehabilitation in India. Given the lack of evidence, there is a natural uncertainty among the health professionals involved in provision of stroke care

For peer review only - http://bmjopen.bmj.com/site/about/guidelines.xhtml

about what intervention could work best for the stroke survivors in an Indian context. The eligibility for participant recruitment in the 'Care for Stroke Intervention' trial will be based on this uncertainty principle. This approach to assess participant eligibility is well established[13].

Setting

Participants will be recruited using the details obtained by the Aarogyasri Trust, which is a trust run by the State Ministry of Health and Family Welfare (MOHFW) to provide insurance for people affected by various health conditions including stroke. The intervention will be provided to the participants at home and they will be asked to use the intervention in their home.

Eligible Participants:

Inclusion Criteria

- Adults (aged ≥18 years)
- Recent diagnosis of first-ever stroke as defined by the WHO [14] (within 3-6 weeks prior to recruitment)
- All kinds of stroke severity (score 1 - 42, according to NIH stroke scale[15-16])
- Stroke survivor medically stable (reaching a point in medical treatment where life-threatening problems following stroke have been brought under control)

For peer review only

For peer review only - http://bmjopen.bmj.com/site/about/guidelines.xhtml

- Post-stroke functional status of the stroke survivor: requiring assistance of at least one person to perform daily activities such as transfers, self-care and mobility (scoring less than the maximum score obtainable in one or more components of the Barthel Index[17])
- Stroke survivor residing with a primary caregiver (family member) at home.

Exclusion Criteria

- Severe cognitive difficulties (scoring >1 in Orientation, Executive function, Inattention and Language components of the NIH Stroke Scale for cognition [18])
- Severe communication problem (scoring >1 in Dysarthria and Best Language component of the NIH Stroke Scale [15-16])
- Severe comorbidities (severe psychiatric illness, hearing loss, vision loss)
- Stroke survivor functionally dependent because of other pre-existing conditions (e.g. amputation, fracture, dementia)
- Stroke survivor without a primary caregiver
- Stroke survivor unwilling/unable to adhere to the study protocol
- Did not meet the training requirements regarding operation of a smartphone

Randomisation

Stroke survivors will receive all-usual treatment for stroke. Participants eligible for inclusion will be identified by a trial investigator. The eligible participants will be

For peer review only - http://bmjopen.bmj.com/site/about/guidelines.xhtml

initially contacted by telephone and they will be contacted in person by the investigator to share, the details about the study to the participant and the identified caregiver. If the participant consents (next of kin if participant is unable to consent) to participate, the informed written consent will be obtained from them.

An entry form will be used to collect baseline information including the contact details of the participant and the identified caregiver. A participant information sheet outlining the study objectives, risks and benefits along with brief information sheet about stroke will be provided to the participant. After completion of this task, information will be forwarded to the independent randomisation centre and the participants will be randomised as soon as these forms are received. Participants eligible for inclusion will be randomised to the intervention or control arm in a 1:1 ratio using a secure, central, password-protected, web-based system. The intervention will be started within 24 hours of randomisation.

Sample size estimation

The two main factors that determine the number of participants needed in this trial are the estimated event rate and the size of the treatment effect. The primary endpoint for the 'Care for Stroke' trial is dependency in activities of daily living measured at six weeks post recruitment.

For peer review only

For peer review only - http://bmjopen.bmj.com/site/about/guidelines.xhtml

Estimated event rate: In a meta-analysis of early supported discharge trial among participants with stroke, 50% of the stroke survivors were either dead or dependent at the end of follow-up and the beneficial effect of the intervention in the treatment group was an odds reduction of 21% of death and dependency[19].

As a non-inferiority one-sided trial, to evaluate the effectiveness of the Smartphone-enabled educational intervention on dependency, I will need approximately 320 participants (160 in each group) to detect a 15% difference in dependency among the participants between the treatment groups with 80% power at the 5 % level of statistical significance and with 20% loss to follow up.

I believe that non-inferiority trials could exclude the possibility of a small degree of inferiority of a new intervention relative to an active control given the sample size. The results of the trial provided by the confidence interval provide a concrete evaluation of the precision actually achieved, superseding any power calculation carried out before the starting the trial.

Intervention

The 'Care for Stroke' intervention will be delivered through a smartphone and it will include information about stroke and the ways to manage post-stroke disabilities.

For peer review only - http://bmjopen.bmj.com/site/about/guidelines.xhtml

The intervention includes 2-3 minutes of several videos in vernacular language organized in five sections. The sections are information about stroke, home-based exercises, functional skills training, activities of daily living, and assistive devices. The intervention will also have an option for the stroke survivor or the identified caregiver to contact the intervention provider for any support.

Intervention Arm

The stroke survivor and their caregiver will receive 45–60 min of training on accessing and use of the intervention (watching videos) via the smartphone. Participants will then be provided with a smartphone preloaded with the 'Care for Stroke' intervention and asked to try it out on their own. Three or more errorless attempts to retrieve any required part of the intervention from the smartphone will be considered successful training. After successful training, participants will be provided with a smartphone loaded with the intervention and will be asked to use this intervention at their discretion at home for six weeks.

The identified caregivers of stroke survivors will be asked to support the stroke survivors as and when necessary to access the intervention from the smartphone. The participants will be telephonically supported minimum once in a week during the intervention period. The telephonic support is essentially to remind and obtain updates from the participants or identified caregivers on utilisation of the

For peer review only - http://bmjopen.bmj.com/site/about/guidelines.xhtml

intervention. This conversation will be documented and the notes will be kept privately in a locked cupboard. The participants in the intervention arm will not be restricted from receiving standard treatment for their stroke.

Control Arm

Standard post stroke rehabilitation: Usual stroke rehabilitation services available for stroke survivors. In general, the standard treatment may include provision of physiotherapy (45minutes to 60 minutes) at home or in a clinic facility for the stroke survivors based on goals set by the therapist.

Outcome Measures

Primary Outcome

The primary outcome measure is the effect of treatment allocation on dependency measured by the modified Rankin Scale [20] (MRS) at six weeks after randomisation. The MRS scale measures the degree of disability or dependence in the activities of daily living of people who have suffered a stroke in six categories. The maximum score a participant can obtain is six (6), which means the participant is dead. A participant without any disability would score zero (0).

Secondary Outcome

Secondary outcome measures will be:

For peer review only - http://bmjopen.bmj.com/site/about/guidelines.xhtml

- Modified Barthel Index [17]
- Modified Caregiver Strain Index [21]
- Quality of Life measured by WHOQOL – BREF [22]
- Use of Health care and Rehabilitation services (Therapy, Hospitalisation and medication, AYUSH, traditional practices etc.)

Costs for rehabilitative care would be collected from both the treatment groups to see whether the Care for Stroke intervention delivered through a smartphone reduces the overall costs of care (cost-effectiveness).

- Direct costs of health care and rehabilitation since the time of stroke
- Indirect costs (A family member giving up paid employment and taking the role of a caregiver, travel costs etc.)

Follow up

An outcome form will be completed at six weeks after randomisation or at death if either happens sooner. A blinded outcome assessor will evaluate the outcomes at baseline and at six weeks. The Stroke Therapy Academic Industry Round Table (STAIRS) strongly recommends a shorter follow-up period to reduce variation in clinical outcome that could occur due to subsequent stroke events that are unrelated to the trial[23]. This will also allow accurate assessment of the outcome and ensure safety of the participants [23].

For peer review only - http://bmjopen.bmj.com/site/about/guidelines.xhtml

Adverse events

Adverse events are very common among acute stroke survivors. Some of the expected adverse events during the trial are

1. Death due to any vascular causes (e.g. myocardial infarction, recurrent stroke),
2. Hospitalization due to post-stroke complications such as infections, brain oedema, seizures, deep vein thrombosis, urinary tract infections, pressure sores and shoulder subluxation, dislocation and fracture.

These events will be documented during follow-up telephone calls and it will be presented to an independent data safety and monitoring committee for unblinded review.

Data Collection and Management

This trial will be centrally coordinated from the trial coordination center (TCC) at the Indian Institute of Public Health (IIPH) Hyderabad. Baseline data will be collected by the investigator and follow-up data will be collected with appropriate translation by an independent blinded outcome assessor on paper forms. These data will be scanned and sent to the TCC for entry into the electronic database. An independent data safety and monitoring committee (DSMC) will be set up to monitor data

For peer review only - http://bmjopen.bmj.com/site/about/guidelines.xhtml

collection and management. A trial steering committee will also be set up to oversee the conduct of the trial.

Analysis

The main analyses will compare all those allocated to the 'Care for Stroke' intervention versus those allocated to the standard treatment group on an 'intention-to-treat' basis, irrespective of whether the participants received the treatment allocated or not. Appropriate effect estimates with a measure of precision (95% confidence interval) will be presented in results of the trial. Subgroup analysis for the primary outcome will be based on stroke severity, location of the Participant (urban/rural), gender and age at stroke. Interaction tests will also be used to test whether the effect of treatment (if any) differs across these subgroups.

Recruitment of participants:

The trial will identify and recruit participants from the stroke insurance records available at the Aarogyasri trust until the sample size is achieved. Currently, the average stroke insurance claim rate through this trust is 10-12 stroke survivors per month. Hence it would take approximately 32-36 months for recruiting the proposed number of participants in this trial.

Conclusion:

For peer review only - http://bmjopen.bmj.com/site/about/guidelines.xhtml

There is a paucity of global evidence on therapy-based stroke rehabilitation, especially in long-term care [24-25]. Available evidence shows that there is no single physical rehabilitation approach that is more effective than combinations of care[26]. Provision of information to stroke survivors and caregivers has been shown to improve functional outcomes[27]. However, the best way to do this is still unclear. Though mHealth strategies have developed various solutions to meet the needs of stroke survivors, the best way to utilise this approach in stroke rehabilitation is also still unclear[28]. There is insufficient evidence for tele-rehabilitation services[29]. This context provides a strong grounding for rigorous research on the 'Care for Stroke' intervention.

Investigating the intervention effectiveness as a priority would provide immense insights for planning, implementation and the potential scalability of the intervention, especially in countries with limited resources. Given the methodological quality of the available evidence [27-29], there is a pressing need to conduct a rigorous (randomized, controlled, sufficiently powered) clinical trial to demonstrate the effectiveness of the 'Care for Stroke' intervention.

Ethics and Dissemination

Ethical approval for this trial has been obtained from the independent institutional research ethics committee at the public health foundation of India (IIPH) Hyderabad.

For peer review only - http://bmjopen.bmj.com/site/about/guidelines.xhtml

Results of this trial will be published in relevant, peer-reviewed, indexed, international journal.

For peer review only

References

1. Mackay J, Mensah G. The atlas of heart disease and stroke. Geneva, Switzerland: WHO. 2004. http://www.who.int/cardiovascular_diseases/en/cvd_atlas_15_burden_stroke.pdf
2. Ferri CP, Schoenborn C, Kaira L, Acosta D, Guerra M, Huang Y, Jacob KS, Llibre Rodriquez JJ, Salas A, Sosa AL, et al. Prevalence of stroke and related burden among older people living in Latin America, India and China. J Neurol Neurosurg Psychiatry 2011;82:1074
3. World report on disability. Geneva, Switzerland: WHO, 2011. http://www.who.int/disabilities/world_report/2011/report/en/
4. Taylor FC, Sureshkumar K. *Stroke in India fact sheet – updated 2012.* SANCD. India. http://www.sancd.org/Updated%20Stroke%20Fact%20sheet%202012.pdf .

For peer review only - http://bmjopen.bmj.com/site/about/guidelines.xhtml

5. Feigin VL, Lawes CM, Bennett DA, Barker-Collo SL, Parag V. Worldwide stroke incidence and early case fatality reported in 56 population-based studies: a systematic review. *Lancet Neurology*. 2009; 8 (4): 355-369.

6. Algurén B, Lundgren-Nilsson Å, Stibrant-Sunnerhagen K. Facilitators and barriers of stroke survivors in the early-post stroke phase. Disability and Rehabilitation, 2009; 31(19): 1584–1591.

7. WHO. *The International Classification of Functioning, Disability and Health*. World Health Organization. Geneva, Switzerland: WHO, 2001.

8. Sureshkumar K, Murthy GVS, Munuswamy S, S. Goenka and H Kuper. 'Care for Stroke' a web-based, Smartphone-enabled educational intervention for management of physical disabilities following stroke: Feasibility in the Indian context. *BMJ Innovations* 2015; 1 127–36.

9. Peter Craig, Mark Petticrew, Developing and evaluating complex interventions: Reflections on the 2008 MRC guidance. *International Journal of Nursing Studies.* 2013; 50 (5): 585-87.

10. Craig P. Foreword. In: Richards D, Rahm Hallberg I. *Complex interventions in health: an overview of research methods.* Routledge, 2015.

11. Sureshkumar K, Murthy GVS, Natarajan S, Naveen C, Goenka S, Kuper H. Evaluation of the feasibility and acceptability of the 'Care for Stroke' intervention in India, a smartphone-enabled, carer-supported, educational

For peer review only - http://bmjopen.bmj.com/site/about/guidelines.xhtml

intervention for management of disability following stroke. BMJ Open 2016;6:e009243. doi:10.1136/bmjopen-2015009243

12. Bowen S, Zwi AB. Pathways to "Evidence-Informed" Policy and Practice: A Framework for Action. *PLoS Medicine*. 2005;2(7):e166. doi:10.1371/journal.pmed.0020166.
13. Peto R, Baigent C. Trials: the next 50 years : Large scale randomised evidence of moderate benefits . *BMJ : British Medical Journal*. 1998;317(7167):1170-1171.
14. WHO MONICA Project Investigators. The World Health Organization MONICA Project (Monitoring trends and determinants in cardiovascular disease). *J Clin Epidemiol*. 1988; (41): 105-114.
15. Department of Health and Human Services. The National Institute of Neurological Disorders and Stroke (NINDS). *NIH Stroke Scale Training, Part 2. Basic Instruction*. 2010. https://archive.org/details/gov.hhs.ninds.stroke.1.2 (accessed 3 August 2010).
16. Ver Hage A. The NIH stroke scale: a window into neurological status. Nurse.Com; *Nursing Spectrum* (Greater Chicago). 2011; 24 (15):44-49.
17. Mahoney F. Barthel D. Functional evaluation: the Barthel Index. *Md Med J*.1965; 14:61–65.
18. Cumming TB, Blomstrand C, Bernhardt J, Linden T. The NIH stroke scale can establish cognitive function after stroke. *Cerebrovasc Dis*. 2010; 30(1):7-14.

19. Langhorne P, Taylor G, Murray G. Dennise M, Anderson C, Bautz Holter E, Dey P, Indredavik B, Mayo N, Power M, et al. Early supported discharge services for stroke Participants: a meta-analysis of individual Participants' data. Lancet. 2005; 365(9458):501–6.

20. Rankin L. Cerebral vascular accidents in Participants over the age of 60. II. Prognosis. *Scott Med J.* 1957; 2: 200-215.

21. Thornton, M., & Travis, S.S. Analysis of the reliability of the Modified Caregiver Strain Index. The Journal of Gerontology, Series B, 2003. Psychological Sciences and Social Sciences, 58B(2), S127-132.

22. World Health Organization's. Quality of Life group: WHOQOL-sBREF Introduction. Administration and Scoring. Field Trial version. 1996

23. Aziz NA, Leonardi-Bee J, Phillips MF, Gladman JR, Legg L, Walker MF. Therapy-based rehabilitation services for Participants living at home more than one year after stroke. *Cochrane Database of Systematic Reviews.* 2008, Issue 2. Art. No.: CD005952. DOI: 10.1002/14651858.CD005952.pub2.

24. OutParticipant Service Trialists. Therapy-based rehabilitation services for stroke Participants at home. *Cochrane Database of Systematic Reviews.* 2003, Issue 1. Art. No.: CD002925. DOI: 10.1002/14651858.CD002925.

25. Turner-Stokes L, Nair A, Sedki I, Disler PB, Wade DT. Multi-disciplinary rehabilitation for acquired brain injury in adults of working age. *Cochrane*

Database of Systematic Reviews. 2005, Issue 3. Art. No.: CD004170. DOI: 10.1002/14651858.CD004170.pub2.

26. Forster A, Brown L, Smith J, House A, Knapp P, Wright JJ, Young J. Information provision for stroke Participants and their caregivers. *Cochrane Database of Systematic Reviews.* 2012, Issue 11. Art. No.: CD001919. DOI: 10.1002/14651858.CD001919.pub3.
27. Laver KE, Schoene D, Crotty M, George S, Lannin NA, Sherrington C. Telerehabilitation services for stroke. *Cochrane Database of Systematic Reviews.* 2013, Issue 12. Art. No.: CD010255. DOI: 10.1002/14651858.CD010255.pub2.
28. Laver KE, George S, Thomas S, Deutsch JE, Crotty M. Virtual reality for stroke rehabilitation. *Cochrane Database of Systematic Reviews.* 2015, Issue 2. Art. No.: CD008349. DOI: 10.1002/14651858.CD008349.pub3.

Authors' Contributions

K Sureshkumar (SK) conceived, designed and drafted the manuscript. Prof GVS Murthy and Dr Hannah Kuper played a crucial role in conception of the research study and provided substantial guidance in designing and conducting evaluation.

Funding

This work was funded and supported **The Wellcome Trust DBT India Alliance.** Grant Code: IA/CPHE/16/1/502650

For peer review only - http://bmjopen.bmj.com/site/about/guidelines.xhtml

Competing Interests

The authors declare that they have no competing interests, financial or non-financial.

Acknowledgement

We thank The Wellcome-trust-DBT India Alliance for funding the research study. We thank the independent institutional research ethics committee of the PHFI-Indian Institute of Public Health - Hyderabad for granting scientific and ethics approval to conduct this research study. We thank the consultants from Suchir softech and Selva photography for developing the software application and digitization of the content of the intervention.

Figure Legends

1. Figure – 1: Flow Chart of the Care for Stroke Trial

For peer review only

For peer review only - http://bmjopen.bmj.com/site/about/guidelines.xhtml

For peer review only

Figure – 1: Flow Chart of the Care for Stroke Trial

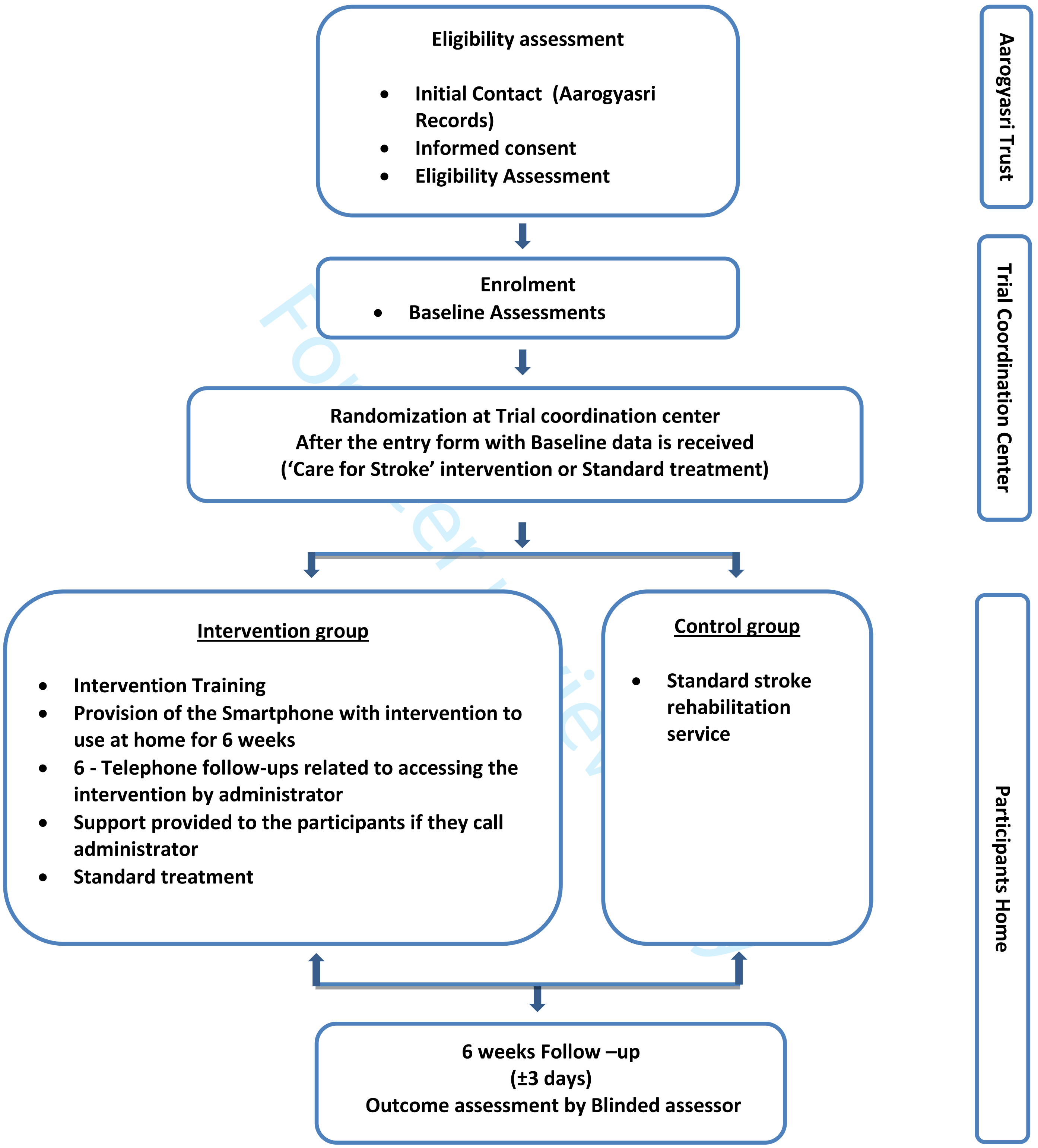

TIDieR
Template for Intervention Description and Replication

The TIDieR (Template for Intervention Description and Replication) Checklist*:

Information to include when describing an intervention and the location of the information

Item number	Item	Where located ** Primary paper (page or appendix number)	Other † (details)
	**BRIEF NAME**		
**1.**	Provide the name or a phrase that describes the intervention.	1, 8, 14	
	**WHY**		
**2.**	Describe any rationale, theory, or goal of the elements essential to the intervention.	6,7,8	
	**WHAT**		
**3.**	Materials: Describe any physical or informational materials used in the intervention, including those provided to participants or used in intervention delivery or in training of intervention providers. Provide information on where the materials can be accessed (e.g. online appendix, URL).	8, 14	
**4.**	Procedures: Describe each of the procedures, activities, and/or processes used in the intervention, including any enabling or support activities.	8, 14	
	**WHO PROVIDED**		
**5.**	For each category of intervention provider (e.g. psychologist, nursing assistant), describe their expertise, background and any specific training given.	14	
	**HOW**		
**6.**	Describe the modes of delivery (e.g. face-to-face or by some other mechanism, such as internet or telephone) of the intervention and whether it was provided individually or in a group.	13, 14, 15	
	**WHERE**		
**7.**	Describe the type(s) of location(s) where the intervention occurred, including any necessary infrastructure or relevant features.	10	

TIDieR checklist

For peer review only

	**WHEN and HOW MUCH**	14	
**8.**	Describe the number of times the intervention was delivered and over what period of time including the number of sessions, their schedule, and their duration, intensity or dose.	______________	______________
	**TAILORING**	7 , 8	
**9.**	If the intervention was planned to be personalised, titrated or adapted, then describe what, why, when, and how.	______________	______________
	**MODIFICATIONS**	N /A	
**10.‡**	If the intervention was modified during the course of the study, describe the changes (what, why, when, and how).	______________	______________
	**HOW WELL**	14	
**11.**	Planned: If intervention adherence or fidelity was assessed, describe how and by whom, and if any strategies were used to maintain or improve fidelity, describe them.	______________	______________
**12.‡**	Actual: If intervention adherence or fidelity was assessed, describe the extent to which the intervention was delivered as planned.	___14_________	______________

** **Authors** - use N/A if an item is not applicable for the intervention being described. **Reviewers** – use '?' if information about the element is not reported/not sufficiently reported.

† If the information is not provided in the primary paper, give details of where this information is available. This may include locations such as a published protocol or other published papers (provide citation details) or a website (provide the URL).

‡ If completing the TIDieR checklist for a protocol, these items are not relevant to the protocol and cannot be described until the study is complete.

* We strongly recommend using this checklist in conjunction with the TIDieR guide (see *BMJ* 2014;348:g1687) which contains an explanation and elaboration for each item.

* The focus of TIDieR is on reporting details of the intervention elements (and where relevant, comparison elements) of a study. Other elements and methodological features of studies are covered by other reporting statements and checklists and have not been duplicated as part of the TIDieR checklist. When a **randomised trial** is being reported, the TIDieR checklist should be used in conjunction with the CONSORT statement (see www.consort-statement.org) as an extension of **Item 5 of the CONSORT 2010 Statement.** When a **clinical trial protocol** is being reported, the TIDieR checklist should be used in conjunction with the SPIRIT statement as an extension of **Item 11 of the SPIRIT 2013 Statement** (see www.spirit-statement.org). For alternate study designs, TIDieR can be used in conjunction with the appropriate checklist for that study design (see www.equator-network.org).

TIDieR checklist

For peer review only

BMJ Open

Protocol for a randomised controlled trial to evaluate the effectiveness of the 'care for stroke' intervention in India; a smartphone-enabled, carer-supported, educational intervention for management of disabilities following stroke

Journal:	*BMJ Open*
Manuscript ID	bmjopen-2017-020098.R1
Article Type:	Protocol
Date Submitted by the Author:	08-Feb-2018
Complete List of Authors:	Sureshkumar, K; Public Health Foundation of India, Indian Institute of Public Health Hyderabad SACDIR; London School of Hygiene and Tropical Medicine, International Centre for Evidence in Disability Department of clinical research<br>Murthy, GVS; Public Health Foundation of India, , Indian Institute of Public Health Hyderabad SACDIR; London School of Hygiene and Tropical Medicine, International Centre for Eye Health<br>Kuper, Hannah; The London School of Hygiene & Tropical Medicine, Clinical Research
**Primary Subject Heading**:	Public health
Secondary Subject Heading:	Rehabilitation medicine, Neurology, Health services research, Evidence based practice, Health policy
Keywords:	Clinical trials < THERAPEUTICS, Stroke < NEUROLOGY, Disability, mHealth, REHABILITATION MEDICINE

SCHOLARONE™
Manuscripts

Protocol for a randomised controlled trial to evaluate the effectiveness of the 'care for stroke' intervention in India; a smartphone-enabled, carer-supported, educational intervention for management of disabilities following stroke

K Sureshkumar[1,3], GVS Murthy[1,3], Hannah Kuper[3]

Corresponding Author:

Sureshkumar Kamalakannan

Indian Institute of Public Health - Hyderabad

Plot No: 1 ANV Arcade, Amar Cooperative Society, Kavuri Hills, Madhapur Hyderabad – 500081, Email: suresh.kumar@iiphh.org

Phone: +91 9676333412, +91 9840772381.

Authors

1. Public Health Foundation of India, Indian institute of Public Health - Hyderabad, South Asia Centre for Disability, Inclusive Development and Research (SACDIR) Plot 1 ANV Arcade, Amar Cooperative Society, Kavuri Hills, Madhapur, Hyderabad, Telangana, 500081. suresh.kumar@iiphh.org
2. Public Health Foundation of India, Indian institute of Public Health - Hyderabad, South Asia Centre for Disability, Inclusive Development and Research (SACDIR) Plot 1 ANV Arcade, Amar Cooperative Society, Kavuri Hills, Madhapur, Hyderabad, Telangana, 500081. murthy.gvs@iiphh.org
3. International Centre for Evidence in Disability, Department of Clinical Research, London School of Hygiene and Tropical Medicine, Keppel Street, London, WC1E 7HT. Hannah.Kuper@lshtm.ac.uk

For peer review only

For peer review only - http://bmjopen.bmj.com/site/about/guidelines.xhtml

Keywords

1. Clinical Trial
2. Stroke
3. Disability
4. Mhealth
5. Rehabilitation
6. Clinical effectiveness

Word Count: Manuscript: - 2769

For peer review only

For peer review only - http://bmjopen.bmj.com/site/about/guidelines.xhtml

Abstract:

Introduction: The rising prevalence of stroke and stroke-related disability witnessed globally over the past decades may cause an overwhelming demand for rehabilitation services. This situation is of concern for low and middle income countries (LMIC) like India where the resources for rehabilitation are often limited. Recently, a smartphone-enabled carer-supported educational intervention for management of physical disabilities following stroke was developed in India. It was found feasible and acceptable, but evidence of effectiveness is lacking. Hence as a step forward, this study intends to evaluate clinical effectiveness of the intervention through a randomized controlled trial.

Methods: The objective of the study is to evaluate whether the 'Care for Stroke' intervention is clinically and cost effective for the reduction of dependency in activities of daily living among stroke survivors in an India setting. This study is designed as a randomised controlled trial comparing people who received the intervention to those receiving standard care. The trial will be pragmatic, and outcome assessor-blinded. The primary outcome for the study is dependency in daily living measured by the Modified Rankin Scale. A total of 234 adult stroke survivors who fulfil the eligibility criteria will be randomised to receive either 'Care for Stroke' intervention or standard treatment and will be followed up for six weeks.

For peer review only - http://bmjopen.bmj.com/site/about/guidelines.xhtml

Analysis: The main analyses will compare participants allocated to the 'Care for Stroke' intervention versus those allocated to the standard treatment group on an 'intention-to-treat' basis, irrespective of whether the participants received the treatment allocated or not. The dichotomised MRS scores (0-3 and 4-6) in both the groups will be used to calculate the effect estimates with a measure of precision (95% confidence interval) and presented in the results of the trial.

Ethics and Dissemination: The Indian Institute of Public Health-Hyderabad / Public Health Foundation of India – Independent Institutional Ethics Committee and the ethics committee of the London School of Hygiene & Tropical Medicine. Dissemination will be through peer-reviewed publications.

Registration Details: Clinical Trial Registry of India CTRI/2017/07/009014.

For peer review only

For peer review only - http://bmjopen.bmj.com/site/about/guidelines.xhtml

Strengths and Limitations of the study:

1. It is a randomized controlled trial protocol and the trial is rigorously designed.
2. The data collection tools and methods have been pilot-tested in the study setting.
3. The follow-up duration is not long.
4. Recruitment of participants is expected to be time consuming, and so the study duration is long.

For peer review only

For peer review only - http://bmjopen.bmj.com/site/about/guidelines.xhtml

Introduction

Globally around 15 million people suffer from stroke each year and a quarter of them experience permanent disability[1]. Much of this burden is borne by Low and Middle Income Countries (LMICs) [2]. The increase in prevalence of stroke[3] and consequently of stroke-related disability may cause an overwhelming demand for rehabilitation services worldwide[3]. This situation is especially of concern for LMICs like India where the resources for rehabilitation are often limited[3].

Stroke is one of the leading causes of death and disability in India. Given the paucity of data on stroke in India, a systematic review of population-based studies on stroke in India was conducted. Studies included in this review showed that the crude stroke prevalence during the past two decades in India ranged from 44/100,000 persons to 559/100,000 persons, [4] and the cumulative incidence of stroke in India ranged from 105-152/100,000 person per year[4]. These estimates on stroke incidence and prevalence are found to be higher than those reported from High Income Countries[5.] The growing burden of stroke-related disability and the unmet need for rehabilitation following stroke in India poses a major public health challenge.

There is a paucity of global evidence on the effectiveness of therapy-based stroke rehabilitation, especially in long-term care [6-7]. Available evidence shows that there is no single physical rehabilitation approach that is more effective than combinations of

For peer review only - http://bmjopen.bmj.com/site/about/guidelines.xhtml

care[8]. Provision of information to stroke survivors and caregivers has been shown to improve functional outcomes[9]. However, the best way to do this is still unclear. Recently, mHealth options are rising substantially and mobile technology has been substantially used to communicate for health-related reasons. Though mHealth strategies have developed various solutions to meet the needs of stroke survivors, the best way to utilise this approach in stroke rehabilitation is also still unclear[10]. There is insufficient evidence for tele-rehabilitation services[11]. This context provides a strong grounding for the development of cost-effective multi-dimensional stroke rehabilitation interventions to meet the demands of the stroke survivors. In the absence of organised stroke care services, and with the limited resources available for rehabilitation, a comprehensive approach to address the growing burden of stroke-related disability in India becomes pertinent[12]. This approach could be pivotal in integrating various strategies for rehabilitation[3] (Educational, Community-based rehabilitation, digital technology, Self/Supported management etc.). It could also be useful for targeting the full range of impacts of stroke, including on impairments, activity limitations and participation restriction, as outlined in the 'Biopsychosocial conceptualization of disability framework' for the intervention, as proposed by the ICF[13].

For peer review only - http://bmjopen.bmj.com/site/about/guidelines.xhtml

A smartphone-enabled carer-supported educational intervention was developed by our group for the management of physical disabilities following stroke in India[14]. This intervention was named as 'Care for Stroke'. It was developed using the systematic approach to development and evaluation of complex interventions, as recommended by the Medical Research Council (MRC) in the U.K. [15-16]. We intended to bridge the gaps in access to stroke services through this innovative intervention which optimises relevant public health practice with the support from mobile devices such as smartphones, personal digital assistants and other wireless devices[17]. To the best of our knowledge, there is no other stroke rehabilitation intervention enabled through mHealth platforms that are available and relevant to India.

The intervention was evaluated for its feasibility and acceptability in an Indian context[18]. The intervention includes information about stroke and the ways to manage physical disability following stroke. It contains a practical demonstration of functional post-stroke exercises to acquire the functional abilities necessary to perform everyday tasks, adaptive techniques to perform one's own daily activities independently and a specific section on assistive devices that could enable participation of the stroke survivors in their daily tasks[14]. Findings from the pilot-testing showed that the 'Care for Stroke' intervention was feasible and acceptable in the Indian context[18]. About 95% of the stroke survivors and all the caregivers (100%)

For peer review only - http://bmjopen.bmj.com/site/about/guidelines.xhtml

rated the intervention as "excellent", based on it's a) overall credibility, b) feasibility and c) user-friendliness[18].

However, feasibility and acceptability alone will not be sufficient to inform implementation and scalability[16]. Nor will it be enough in order to advocate for change in policy towards implementation of an intervention[19]. Investigating the intervention clinical and cost effectiveness will provide insights for planning, implementation and the potential scalability of the intervention, especially in countries with limited resources. Given the methodological quality of the available evidence [9-11], there is a pressing need to conduct a rigorous (randomized, controlled, sufficiently powered) clinical trial to demonstrate the effectiveness of the 'Care for Stroke' intervention.

Objective:

The objective of the randomised controlled trial is to evaluate whether the 'Care for Stroke' intervention is effective for the reduction of dependency in activities of daily living among stroke survivors compared to people receiving standard treatment in an India setting. The primary outcome for the study is disability measured by the Modified Rankin Scale.

For peer review only - http://bmjopen.bmj.com/site/about/guidelines.xhtml

Methods:

Overview

This trial will be a pragmatic, randomised, outcome assessor-blinded trial to quantify the effectiveness of the Care for Stroke Intervention on reducing dependency in activities of daily living following stroke. A total of 234 adult stroke survivors who fulfil the eligibility criteria will be randomised to receive either 'Care for Stroke' intervention or standard treatment and will be followed for six weeks. The flow chart of the entire trial process is provided in figure - 1.

Pragmatic design and the uncertainty principle

The effectiveness of the intervention in routine practice can be assessed using the pragmatic trial design. Until now, there is no evidence for effectiveness of stroke rehabilitation interventions that is unidisciplinary, led by a physician, neurologist or a physiotherapist alone[12]. However, a physiotherapist or physician-driven unidisciplinary rehabilitation is what is commonly practiced in the context of stroke rehabilitation in India[12]. Given the lack of evidence, there is a natural uncertainty among the health professionals involved in provision of stroke care about what intervention could work best for the stroke survivors in an Indian context. The eligibility for participant recruitment in the 'Care for Stroke Intervention' trial will be based on this uncertainty principle. This approach to assess participant eligibility is well established[20].

For peer review only - http://bmjopen.bmj.com/site/about/guidelines.xhtml

Setting

Participant Recruitment

Participants will be identified using their contact details from treatment records for their first ever stroke. These details for stroke survivors in India exist in two places. Participant diagnosis and details can be collected from the hospital records from which an individual received treatment for his/her stroke. It is also available at the government health insurance department where the cost of the treatment for stroke is covered by this insurance department. Hence participants will be identified through both these options. The identified participants will be contacted at their home for consent and recruitment. The intervention will be provided to the participants at home and they will be asked to use the intervention in their home.

Eligible Participants:

Inclusion Criteria

- Adults (aged ≥18 years)
- Recent diagnosis of first-ever stroke as defined by the WHO [21]
- Any level of stroke severity (score 1 - 42, according to NIH stroke scale[22-23])
- Stroke survivor medically stable (reaching a point in medical treatment where life-threatening problems following stroke have been brought under control)

For peer review only - http://bmjopen.bmj.com/site/about/guidelines.xhtml

- Post-stroke functional status of the stroke survivor: requiring assistance of at least one person to perform daily activities such as transfers, self-care and mobility (i.e. scoring less than the maximum score obtainable in one or more components of the Barthel Index[24])
- Stroke survivor residing with a primary caregiver (family member) at home.

Exclusion Criteria

- Severe cognitive difficulties (scoring >1 in Orientation, Executive function, Inattention and Language components of the NIH Stroke Scale for cognition [25])
- Severe communication problem (scoring >1 in Dysarthria and Best Language component of the NIH Stroke Scale [22-23])
- Stroke survivor functionally dependent because of other pre-existing conditions (e.g. amputation, fracture, dementia)
- Stroke survivor without a primary caregiver
- Stroke survivor unwilling/unable to adhere to the study protocol
- Stroke survivors who did not meet the training requirements regarding operation of a smartphone

Randomisation

For peer review only - http://bmjopen.bmj.com/site/about/guidelines.xhtml

Stroke survivors will receive all-usual treatment for stroke. Participants eligible for inclusion will be identified by a trial investigator. The eligible participants will be initially contacted by telephone and they will be visited in person at their home by the investigator to share the details about the study to the participant and the identified caregiver. A participant information sheet outlining the study objectives, risks and benefits along with brief information sheet about stroke will be provided to the participant. Written informed consent for participation in the intervention will be sought from all participants or from the next of kin if the participant is unable to consent.

An entry form will be used to collect baseline information including the contact details of the participant and the identified caregiver. This information will be forwarded to the independent randomisation centre and the participants eligible for inclusion will be randomised to the intervention or control arm in a 1:1 ratio using a secure, central, password-protected, web-based system. The intervention will be started within 24 hours of randomisation.

Sample size estimation

The two main factors that determine the number of participants needed in this trial are the estimated event rate and the size of the treatment effect. The primary

For peer review only - http://bmjopen.bmj.com/site/about/guidelines.xhtml

outcome for the 'Care for Stroke' trial is dependency in activities of daily living measured at six weeks post recruitment.

Estimated event rate**:** In a meta-analysis of early supported discharge trial among participants with stroke, 50% of the stroke survivors were either dead or dependent at the end of follow-up and the beneficial effect of the intervention in the treatment group was an odds reduction of 21% of death and dependency[26].

As a non-inferiority one-sided trial, to evaluate the effectiveness of the Smartphone-enabled educational intervention on dependency, there will be a requirement of approximately 234 participants (117 in each group) to detect a 20% difference in dependency among the participants between the treatment groups with 80% power at the 5 % level of statistical significance and with 20% loss to follow up.

A non-inferiority trial could exclude the possibility of a small degree of inferiority of a new intervention relative to an active control given the sample size. The results of the trial provided by the confidence interval will allow concrete evaluation of the precision actually achieved, superseding any power calculation carried out before the starting the trial.

Intervention

For peer review only - http://bmjopen.bmj.com/site/about/guidelines.xhtml

The 'Care for Stroke' intervention will be delivered through a smartphone and it will include information about stroke and the ways to manage post-stroke disabilities. The intervention includes 2-3 minutes of 60 videos in vernacular language organized in five sections. The sections are: 1) information about stroke, 2) home-based exercises, 3) functional skills training, 4) activities of daily living, and 5) assistive devices. The intervention will be self-directed, with participants seeking information in the different categories as they require. The intervention will also have an option for the stroke survivor or the identified caregiver to contact the intervention provider for any technical support in accessing the intervention through Smartphone.

Intervention Arm

The stroke survivor and their caregiver will receive 45–60 min of training on accessing and use of the intervention (watching videos) via the smartphone. Participants will then be provided with a smartphone preloaded with the 'Care for Stroke' intervention and asked to try it out on their own. Three or more errorless attempts to retrieve any required part of the intervention from the smartphone will be considered successful training. After successful training, participants will be provided with a smartphone loaded with the intervention and will be asked to use this intervention at their discretion at home over a six week period.

For peer review only - http://bmjopen.bmj.com/site/about/guidelines.xhtml

The identified caregivers of stroke survivors will be asked to support the stroke survivors as and when necessary to access the intervention from the smartphone. The participants will be telephonically supported at least once in a week during the intervention period. The telephonic support is essentially to remind and obtain updates from the participants or identified caregivers on utilisation of the intervention. A summary of this conversation will be documented and the notes will be kept privately in a locked cupboard. The participants in the intervention arm will not be restricted from receiving standard treatment for their stroke.

Control Arm

Participants in the control arm will receive standard post stroke rehabilitation services. In general, the standard treatment may include provision of physiotherapy (45 minutes to 60 minutes) at home or in a clinic facility for the stroke survivors based on goals set by the specific therapist or a rehabilitation team.

Outcome Measures

Primary Outcome

The primary outcome measure is dependency in activities of daily living and will be measured by the Modified Rankin Scale [27] (MRS) at baseline and at six weeks after randomisation. The MRS scale measures the degree of disability or dependence in the activities of daily living of people who have suffered a stroke in six categories.

For peer review only - http://bmjopen.bmj.com/site/about/guidelines.xhtml

The scores range from zero (no symptoms) to a maximum of six (dead). A dichotomous approach to outcome analysis will be used. Participants' scores will be categorised into MRS scores of 0-3 and 4-6.

Secondary Outcome

Secondary outcome measures will be:

- Modified Barthel Index [24]
- Modified Caregiver Strain Index [28]
- Quality of Life measured by WHOQOL – BREF [29]
- Use of Health care and Rehabilitation services (Therapy, Hospitalisation and medication, AYUSH, traditional practices etc.)

This information will be collected through questionnaire at baseline and after 6 weeks. The Smartphone application has an inbuilt monitoring mechanism where the usage of the intervention by the participants will be tracked.

Costs for rehabilitative care will be collected from participants both in the treatment groups to see whether the Care for Stroke intervention delivered through a smartphone reduces the overall costs of care (cost-effectiveness).

- Direct costs of health care and rehabilitation since the time of stroke
- Indirect costs (A family member giving up paid employment and taking the role of a caregiver, travel costs etc.)

For peer review only - http://bmjopen.bmj.com/site/about/guidelines.xhtml

Follow up

An outcome form will be completed at six weeks after randomisation or at death, if either happens sooner. A blinded outcome assessor will evaluate all the outcomes (primary and secondary) at baseline and at six weeks. A relatively short follow-up period has been selected as The Stroke Therapy Academic Industry Round Table (STAIRS) strongly recommends a shorter follow-up period to reduce variation in clinical outcome that could occur due to subsequent stroke events that are unrelated to the trial[24]. This will also allow accurate assessment of the outcome [30].

Adverse events

Adverse events are very common among acute stroke survivors. Some of the expected adverse events during the trial are

1. Death due to any vascular causes (e.g. myocardial infarction, recurrent stroke),
2. Hospitalization due to post-stroke complications such as infections, brain oedema, seizures, deep vein thrombosis, urinary tract infections, pressure sores and shoulder subluxation, dislocation and fracture.
3. Occurrence of secondary stroke.

For peer review only - http://bmjopen.bmj.com/site/about/guidelines.xhtml

These events will be documented during follow-up telephone calls and it will be presented to an independent data safety and monitoring committee for unblinded review.

Data Collection and Management

This trial will be centrally coordinated from the trial coordination center (TCC) at the Indian Institute of Public Health (IIPH) Hyderabad. Baseline data will be collected by the investigator and follow-up data will be collected with appropriate translation by an independent blinded outcome assessor on paper forms. These data will be securely scanned and sent to the TCC for entry into the password protected secured electronic database. An independent data safety and monitoring committee (DSMC) will be set up to monitor data collection and management. A trial steering committee will also be set up to oversee the conduct of the trial.

For peer review only

Analysis

The main analyses will compare all those allocated to the 'Care for Stroke' intervention versus those allocated to the standard treatment group on an 'intention-to-treat' basis, irrespective of whether the participants received the treatment allocated or not. Appropriate effect estimates with a measure of precision (95%

For peer review only - http://bmjopen.bmj.com/site/about/guidelines.xhtml

confidence interval) will be presented in results of the trial. Subgroup analysis for the primary outcome will be based on stroke severity, location of the participant (urban/rural), gender and age at stroke. Interaction tests will also be used to test whether the effect of treatment (if any) differs across these subgroups.

For peer review only

Recruitment of participants:

The trial will identify and recruit participants from the hospital records as well as stroke insurance records available at the Aarogyasri trust until the sample size is achieved. Currently, the average stroke insurance claim rate through this trust is 10-12 stroke survivors per month. Hence it would take approximately 32-36 months for recruiting the proposed number of participants in this trial.

Ethics and Dissemination

Ethical approval for this trial has been obtained from the independent institutional research ethics committee at the public health foundation of India (IIPH) Hyderabad. Results of this trial will be published in relevant, peer-reviewed, indexed, international journal.

References

For peer review only - http://bmjopen.bmj.com/site/about/guidelines.xhtml

1. Global Burden of Disease Collaborative Network. Global Burden of Disease Study 2016 (GBD 2016) Disability-Adjusted Life Years and Healthy Life Expectancy 1990-2016. Seattle, United States: Institute for Health Metrics and Evaluation (IHME), 2017.
2. Ferri CP, Schoenborn C, Kaira L, Acosta D, Guerra M, Huang Y, Jacob KS, Llibre Rodriquez JJ, Salas A, Sosa AL, et al. Prevalence of stroke and related burden among older people living in Latin America, India and China. J Neurol Neurosurg Psychiatry 2011;82:1074
3. World report on disability. Geneva, Switzerland: WHO, 2011. http://www.who.int/disabilities/world_report/2011/report/en/
4. Kamalakannan Sureshkumar, Gudlavalleti Aashrai S. V., Gudlavalleti Venkata S. Murthy, Goenka Shifalika, Kuper Hannah. Incidence & prevalence of stroke in India: A systematic review. 2017; 146 (2): 175-185
5. Feigin VL, Lawes CM, Bennett DA, Barker-Collo SL, Parag V. Worldwide stroke incidence and early case fatality reported in 56 population-based studies: a systematic review. *Lancet Neurology*. 2009; 8 (4): 355-369.
6. OutParticipant Service Trialists. Therapy-based rehabilitation services for stroke Participants at home. *Cochrane Database of Systematic Reviews*. 2003, Issue 1. Art. No.: CD002925. DOI: 10.1002/14651858.CD002925.
7. Turner-Stokes L, Nair A, Sedki I, Disler PB, Wade DT. Multi-disciplinary rehabilitation for acquired brain injury in adults of working age. *Cochrane*

For peer review only - http://bmjopen.bmj.com/site/about/guidelines.xhtml

Database of Systematic Reviews. 2005, Issue 3. Art. No.: CD004170. DOI: 10.1002/14651858.CD004170.pub2.

8. Forster A, Brown L, Smith J, House A, Knapp P, Wright JJ, Young J. Information provision for stroke Participants and their caregivers. *Cochrane Database of Systematic Reviews.* 2012, Issue 11. Art. No.: CD001919. DOI: 10.1002/14651858.CD001919.pub3.
9. Sarfo FS, Ovbiagele B. Mobile health for stroke: a promising concept for research and practice. *mHealth*. 2017; 3: 4. doi:10.21037/mhealth.2017.02.01.
10. Laver KE, Schoene D, Crotty M, George S, Lannin NA, Sherrington C. Telerehabilitation services for stroke. *Cochrane Database of Systematic Reviews.* 2013, Issue 12. Art. No.: CD010255. DOI: 10.1002/14651858.CD010255.pub2.
11. Laver KE, George S, Thomas S, Deutsch JE, Crotty M. Virtual reality for stroke rehabilitation. *Cochrane Database of Systematic Reviews.* 2015, Issue 2. Art. No.: CD008349. DOI: 10.1002/14651858.CD008349.pub3.
12. Kamalakannan, Sureshkumar et al. Rehabilitation needs of Stroke Survivors after Discharge from Hospital in India. Archives of Physical Medicine and Rehabilitation; 2016. Volume 97, Issue 9, 1526 - 1532.e9.
13. WHO. *The International Classification of Functioning, Disability and Health*. World Health Organization. Geneva, Switzerland: WHO, 2001.
14. Sureshkumar K, Murthy GVS, Munuswamy S, S. Goenka and H Kuper. 'Care for Stroke' a web-based, Smartphone-enabled educational intervention for

management of physical disabilities following stroke: Feasibility in the Indian context. *BMJ Innovations* 2015; 1 127–36.

15. Peter Craig, Mark Petticrew, Developing and evaluating complex interventions: Reflections on the 2008 MRC guidance. *International Journal of Nursing Studies*. 2013; 50 (5): 585-87.
16. Craig P. Foreword. In: Richards D, Rahm Hallberg I. *Complex interventions in health: an overview of research methods.* Routledge, 2015.
17. World Health Organization mHealth: New Horizons for Health through Mobile Technologies: Based on the Findings of the Second Global Survey on eHealth (Global Observatory for eHealth Series, Volume 3) 2011. [2013-0523].
18. Sureshkumar K, Murthy GVS, Natarajan S, Naveen C, Goenka S, Kuper H. Evaluation of the feasibility and acceptability of the 'Care for Stroke' intervention in India, a smartphone-enabled, carer-supported, educational intervention for management of disability following stroke. BMJ Open 2016;6:e009243. doi:10.1136/bmjopen-2015009243
19. Bowen S, Zwi AB. Pathways to "Evidence-Informed" Policy and Practice: A Framework for Action. *PLoS Medicine*. 2005;2(7):e166. doi:10.1371/journal.pmed.0020166.
20. Peto R, Baigent C. Trials: the next 50 years : Large scale randomised evidence of moderate benefits . *BMJ : British Medical Journal*. 1998;317(7167):1170-1171.

For peer review only - http://bmjopen.bmj.com/site/about/guidelines.xhtml

21. WHO MONICA Project Investigators. The World Health Organization MONICA Project (Monitoring trends and determinants in cardiovascular disease). *J Clin Epidemiol.* 1988; (41): 105-114.

22. Department of Health and Human Services. The National Institute of Neurological Disorders and Stroke (NINDS). *NIH Stroke Scale Training, Part 2. Basic Instruction*. 2010. https://archive.org/details/gov.hhs.ninds.stroke.1.2 (accessed 3 August 2010).

23. Ver Hage A. The NIH stroke scale: a window into neurological status. Nurse.Com; *Nursing Spectrum* (Greater Chicago). 2011; 24 (15):44-49.

24. Mahoney F. Barthel D. Functional evaluation: the Barthel Index. *Md Med J.*1965; 14:61–65.

25. Cumming TB, Blomstrand C, Bernhardt J, Linden T. The NIH stroke scale can establish cognitive function after stroke. *Cerebrovasc Dis*. 2010; 30(1):7-14.

26. Langhorne P, Taylor G, Murray G. Dennise M, Anderson C, Bautz Holter E, Dey P, Indredavik B, Mayo N, Power M, et al. Early supported discharge services for stroke Participants: a meta-analysis of individual Participants' data. Lancet. 2005; 365(9458):501–6.

27. Rankin L. Cerebral vascular accidents in Participants over the age of 60. II. Prognosis. *Scott Med J.* 1957; 2: 200-215.

For peer review only - http://bmjopen.bmj.com/site/about/guidelines.xhtml

28. Thornton, M., & Travis, S.S. Analysis of the reliability of the Modified Caregiver Strain Index. The Journal of Gerontology, Series B, 2003. Psychological Sciences and Social Sciences, 58B(2), S127-132.

29. World Health Organization's. Quality of Life group: WHOQOL-sBREF Introduction. Administration and Scoring. Field Trial version. 1996

30. Aziz NA, Leonardi-Bee J, Phillips MF, Gladman JR, Legg L, Walker MF. Therapy-based rehabilitation services for Participants living at home more than one year after stroke. *Cochrane Database of Systematic Reviews.* 2008, Issue 2. Art. No.: CD005952. DOI: 10.1002/14651858.CD005952.pub2.

Authors' Contributions

K Sureshkumar (SK) conceived, designed and drafted the manuscript. Prof GVS Murthy and Dr Hannah Kuper played a crucial role in conception of the research study and provided substantial guidance in designing and conducting evaluation.

Funding

This work was funded and supported **The Wellcome Trust DBT India Alliance.** Grant Code: IA/CPHE/16/1/502650

Competing Interests

The authors declare that they have no competing interests, financial or non-financial.

For peer review only - http://bmjopen.bmj.com/site/about/guidelines.xhtml

Acknowledgement

We thank The Wellcome-trust-DBT India Alliance for funding the research study. We thank the independent institutional research ethics committee of the PHFI-Indian Institute of Public Health - Hyderabad for granting scientific and ethics approval to conduct this research study. We thank the consultants from Suchir softech and Selva photography for developing the software application and digitization of the content of the intervention.

For peer review only

Figure Legends

1. Figure – 1: Flow Chart of the Care for Stroke Trial

For peer review only - http://bmjopen.bmj.com/site/about/guidelines.xhtml

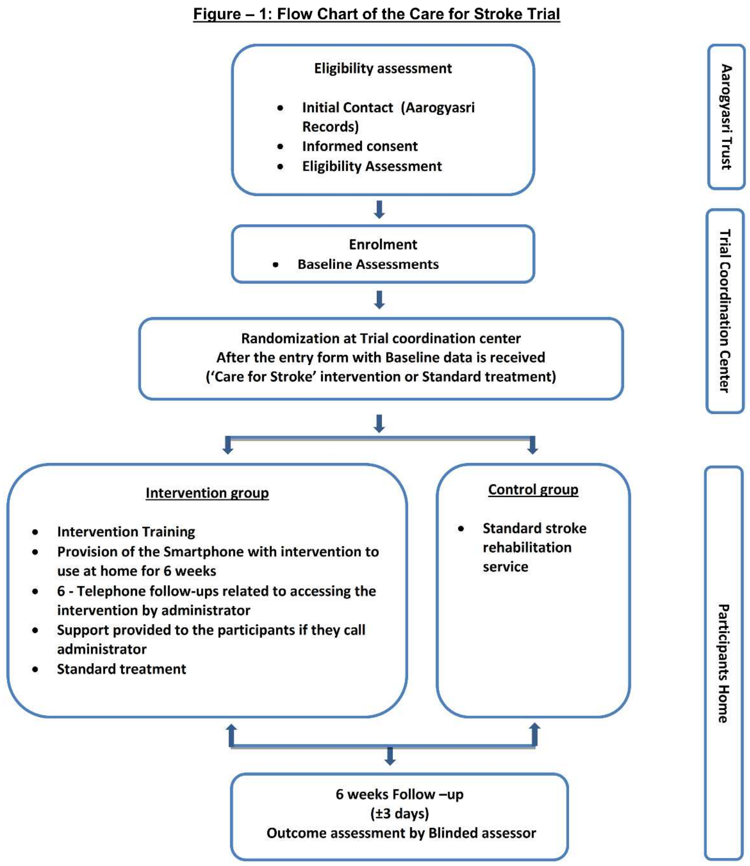

Figure – 1: Flow Chart of the Care for Stroke Trial Process

SPIRIT
STANDARD PROTOCOL ITEMS: RECOMMENDATIONS FOR INTERVENTIONAL TRIALS

SPIRIT 2013 Checklist: Recommended items to address in a clinical trial protocol and related documents*

Section/item	Item No	Description	Addressed on page number
**Administrative information**			
Title	1	Descriptive title identifying the study design, population, interventions, and, if applicable, trial acronym	1
Trial registration	2a	Trial identifier and registry name. If not yet registered, name of intended registry	4
	2b	All items from the World Health Organization Trial Registration Data Set	4
Protocol version	3	Date and version identifier	1
Funding	4	Sources and types of financial, material, and other support	25
Roles and responsibilities	5a	Names, affiliations, and roles of protocol contributors	1
	5b	Name and contact information for the trial sponsor	25
	5c	Role of study sponsor and funders, if any, in study design; collection, management, analysis, and interpretation of data; writing of the report; and the decision to submit the report for publication, including whether they will have ultimate authority over any of these activities	N/A
	5d	Composition, roles, and responsibilities of the coordinating centre, steering committee, endpoint adjudication committee, data management team, and other individuals or groups overseeing the trial, if applicable (see Item 21a for data monitoring committee)	19

For peer review only

**Introduction**			
Background and rationale	6a	Description of research question and justification for undertaking the trial, including summary of relevant studies (published and unpublished) examining benefits and harms for each intervention	6-8
	6b	Explanation for choice of comparators	6-8
Objectives	7	Specific objectives or hypotheses	9
Trial design	8	Description of trial design including type of trial (eg, parallel group, crossover, factorial, single group), allocation ratio, and framework (eg, superiority, equivalence, noninferiority, exploratory)	9,14
**Methods: Participants, interventions, and outcomes**			
Study setting	9	Description of study settings (eg, community clinic, academic hospital) and list of countries where data will be collected. Reference to where list of study sites can be obtained	11
Eligibility criteria	10	Inclusion and exclusion criteria for participants. If applicable, eligibility criteria for study centres and individuals who will perform the interventions (eg, surgeons, psychotherapists)	11
Interventions	11a	Interventions for each group with sufficient detail to allow replication, including how and when they will be administered	14-15
	11b	Criteria for discontinuing or modifying allocated interventions for a given trial participant (eg, drug dose change in response to harms, participant request, or improving/worsening disease)	17
	11c	Strategies to improve adherence to intervention protocols, and any procedures for monitoring adherence (eg, drug tablet return, laboratory tests)	14-15
	11d	Relevant concomitant care and interventions that are permitted or prohibited during the trial	14-15
Outcomes	12	Primary, secondary, and other outcomes, including the specific measurement variable (eg, systolic blood pressure), analysis metric (eg, change from baseline, final value, time to event), method of aggregation (eg, median, proportion), and time point for each outcome. Explanation of the clinical relevance of chosen efficacy and harm outcomes is strongly recommended	16-17
Participant timeline	13	Time schedule of enrolment, interventions (including any run-ins and washouts), assessments, and visits for participants. A schematic diagram is highly recommended (see Figure)	Figure-1

For peer review only - http://bmjopen.bmj.com/site/about/guidelines.xhtml

Sample size	14	Estimated number of participants needed to achieve study objectives and how it was determined, including clinical and statistical assumptions supporting any sample size calculations	13
Recruitment	15	Strategies for achieving adequate participant enrolment to reach target sample size	11

Methods: Assignment of interventions (for controlled trials)

Allocation:

Sequence generation	16a	Method of generating the allocation sequence (eg, computer-generated random numbers), and list of any factors for stratification. To reduce predictability of a random sequence, details of any planned restriction (eg, blocking) should be provided in a separate document that is unavailable to those who enrol participants or assign interventions	12-13
Allocation concealment mechanism	16b	Mechanism of implementing the allocation sequence (eg, central telephone; sequentially numbered, opaque, sealed envelopes), describing any steps to conceal the sequence until interventions are assigned	12-13
Implementation	16c	Who will generate the allocation sequence, who will enrol participants, and who will assign participants to interventions	12-13
Blinding (masking)	17a	Who will be blinded after assignment to interventions (eg, trial participants, care providers, outcome assessors, data analysts), and how	17
	17b	If blinded, circumstances under which unblinding is permissible, and procedure for revealing a participant's allocated intervention during the trial	18

Methods: Data collection, management, and analysis

Data collection methods	18a	Plans for assessment and collection of outcome, baseline, and other trial data, including any related processes to promote data quality (eg, duplicate measurements, training of assessors) and a description of study instruments (eg, questionnaires, laboratory tests) along with their reliability and validity, if known. Reference to where data collection forms can be found, if not in the protocol	17-18
	18b	Plans to promote participant retention and complete follow-up, including list of any outcome data to be collected for participants who discontinue or deviate from intervention protocols	16-17

For peer review only - http://bmjopen.bmj.com/site/about/guidelines.xhtml

Data management	19	Plans for data entry, coding, security, and storage, including any related processes to promote data quality (eg, double data entry; range checks for data values). Reference to where details of data management procedures can be found, if not in the protocol	19
Statistical methods	20a	Statistical methods for analysing primary and secondary outcomes. Reference to where other details of the statistical analysis plan can be found, if not in the protocol	19
	20b	Methods for any additional analyses (eg, subgroup and adjusted analyses)	19
	20c	Definition of analysis population relating to protocol non-adherence (eg, as randomised analysis), and any statistical methods to handle missing data (eg, multiple imputation)	19
**Methods: Monitoring**			
Data monitoring	21a	Composition of data monitoring committee (DMC); summary of its role and reporting structure; statement of whether it is independent from the sponsor and competing interests; and reference to where further details about its charter can be found, if not in the protocol. Alternatively, an explanation of why a DMC is not needed	19
	21b	Description of any interim analyses and stopping guidelines, including who will have access to these interim results and make the final decision to terminate the trial	19
Harms	22	Plans for collecting, assessing, reporting, and managing solicited and spontaneously reported adverse events and other unintended effects of trial interventions or trial conduct	19
Auditing	23	Frequency and procedures for auditing trial conduct, if any, and whether the process will be independent from investigators and the sponsor	19
**Ethics and dissemination**			
Research ethics approval	24	Plans for seeking research ethics committee/institutional review board (REC/IRB) approval	20
Protocol amendments	25	Plans for communicating important protocol modifications (eg, changes to eligibility criteria, outcomes, analyses) to relevant parties (eg, investigators, REC/IRBs, trial participants, trial registries, journals, regulators)	20

For peer review only - http://bmjopen.bmj.com/site/about/guidelines.xhtml

Consent or assent	26a	Who will obtain informed consent or assent from potential trial participants or authorised surrogates, and how (see Item 32)	13
	26b	Additional consent provisions for collection and use of participant data and biological specimens in ancillary studies, if applicable	13
Confidentiality	27	How personal information about potential and enrolled participants will be collected, shared, and maintained in order to protect confidentiality before, during, and after the trial	18
Declaration of interests	28	Financial and other competing interests for principal investigators for the overall trial and each study site	25
Access to data	29	Statement of who will have access to the final trial dataset, and disclosure of contractual agreements that limit such access for investigators	18-19
Ancillary and post-trial care	30	Provisions, if any, for ancillary and post-trial care, and for compensation to those who suffer harm from trial participation	N/A
Dissemination policy	31a	Plans for investigators and sponsor to communicate trial results to participants, healthcare professionals, the public, and other relevant groups (eg, via publication, reporting in results databases, or other data sharing arrangements), including any publication restrictions	20
	31b	Authorship eligibility guidelines and any intended use of professional writers	N/A
	31c	Plans, if any, for granting public access to the full protocol, participant-level dataset, and statistical code	4,20
**Appendices**			
Informed consent materials	32	Model consent form and other related documentation given to participants and authorised surrogates	NO
Biological specimens	33	Plans for collection, laboratory evaluation, and storage of biological specimens for genetic or molecular analysis in the current trial and for future use in ancillary studies, if applicable	N/A

*It is strongly recommended that this checklist be read in conjunction with the SPIRIT 2013 Explanation & Elaboration for important clarification on the items. Amendments to the protocol should be tracked and dated. The SPIRIT checklist is copyrighted by the SPIRIT Group under the Creative Commons "Attribution-NonCommercial-NoDerivs 3.0 Unported" license.

Protocol for a randomised controlled trial to evaluate the effectiveness of the 'care for stroke' intervention in India; a smartphone-enabled, carer-supported, educational intervention for management of disabilities following stroke

Journal:	*BMJ Open*
Manuscript ID	bmjopen-2017-020098.R2
Article Type:	Protocol
Date Submitted by the Author:	03-Apr-2018
Complete List of Authors:	Sureshkumar, K; Public Health Foundation of India, Indian Institute of Public Health Hyderabad SACDIR; London School of Hygiene and Tropical Medicine, International Centre for Evidence in Disability Department of clinical research<br>Murthy, GVS; Public Health Foundation of India, , Indian Institute of Public Health Hyderabad SACDIR; London School of Hygiene and Tropical Medicine, International Centre for Eye Health<br>Kuper, Hannah; The London School of Hygiene & Tropical Medicine, Clinical Research
**Primary Subject Heading**:	Public health
Secondary Subject Heading:	Rehabilitation medicine, Neurology, Health services research, Evidence based practice, Health policy
Keywords:	Clinical trials < THERAPEUTICS, Stroke < NEUROLOGY, Disability, mHealth, REHABILITATION MEDICINE

For peer review only

Protocol for a randomised controlled trial to evaluate the effectiveness of the 'care for stroke' intervention in India; a smartphone-enabled, carer-supported, educational intervention for management of disabilities following stroke

K Sureshkumar[1,3], GVS Murthy[1,3], Hannah Kuper[3]

Corresponding Author:

Sureshkumar Kamalakannan

Indian Institute of Public Health - Hyderabad

Plot No: 1 ANV Arcade, Amar Cooperative Society, Kavuri Hills, Madhapur Hyderabad – 500081, Email: suresh.kumar@iiphh.org

Phone: +91 9676333412, +91 9840772381.

Authors

1. Public Health Foundation of India, Indian institute of Public Health - Hyderabad, South Asia Centre for Disability, Inclusive Development and Research (SACDIR) Plot 1 ANV Arcade, Amar Cooperative Society, Kavuri Hills, Madhapur, Hyderabad, Telangana, 500081. suresh.kumar@iiphh.org
2. Public Health Foundation of India, Indian institute of Public Health - Hyderabad, South Asia Centre for Disability, Inclusive Development and Research (SACDIR) Plot 1 ANV Arcade, Amar Cooperative Society, Kavuri Hills, Madhapur, Hyderabad, Telangana, 500081. murthy.gvs@iiphh.org
3. International Centre for Evidence in Disability, Department of Clinical Research, London School of Hygiene and Tropical Medicine, Keppel Street, London, WC1E 7HT. Hannah.Kuper@lshtm.ac.uk

For peer review only - http://bmjopen.bmj.com/site/about/guidelines.xhtml

Keywords

1. Clinical Trial
2. Stroke
3. Disability
4. Mhealth
5. Rehabilitation
6. Clinical effectiveness

Word Count: Manuscript: - 2769

For peer review only - http://bmjopen.bmj.com/site/about/guidelines.xhtml

Abstract:

Introduction: The rising prevalence of stroke and stroke-related disability witnessed globally over the past decades may cause an overwhelming demand for rehabilitation services. This situation is of concern for low and middle income countries (LMIC) like India where the resources for rehabilitation are often limited. Recently, a smartphone-enabled carer-supported educational intervention for management of physical disabilities following stroke was developed in India. It was found feasible and acceptable, but evidence of effectiveness is lacking. Hence as a step forward, this study intends to evaluate clinical effectiveness of the intervention through a randomized controlled trial.

Methods: The objective of the study is to evaluate whether the 'Care for Stroke' intervention is clinically and cost effective for the reduction of dependency in activities of daily living among stroke survivors in an India setting. This study is designed as a randomised controlled trial comparing people who received the intervention to those receiving standard care. The trial will be pragmatic, and outcome assessor-blinded. The primary outcome for the study is dependency in daily living measured by the Modified Rankin Scale. A total of 266 adult stroke survivors who fulfil the eligibility criteria will be randomised to receive either 'Care for Stroke' intervention or standard treatment and will be followed up for six weeks. The main analyses will compare participants allocated to the 'Care for Stroke'

For peer review only - http://bmjopen.bmj.com/site/about/guidelines.xhtml

intervention versus those allocated to the standard treatment group on an 'intention-to-treat' basis, irrespective of whether the participants received the treatment allocated or not. The dichotomised MRS scores (0-3 and 4-6) in both the groups will be used to calculate the effect estimates with a measure of precision (95% confidence interval) and presented in the results of the trial.

Ethics and Dissemination: The Indian Institute of Public Health-Hyderabad / Public Health Foundation of India – Independent Institutional Ethics Committee and the ethics committee of the London School of Hygiene & Tropical Medicine. Dissemination will be through peer-reviewed publications.

Registration Details: Clinical Trial Registry of India CTRI/2017/07/009014.

For peer review only

For peer review only - http://bmjopen.bmj.com/site/about/guidelines.xhtml

Strengths and Limitations of the study:

1. Effectiveness of the intervention will be established through a randomised controlled trial.
2. The trial protocol was pilot tested and was found feasible.
3. This is the first ever stroke trial in India evaluating a mHealth rehab intervention
4. Stringent inclusion criteria for participant recruitment.
5. The duration of follow-up in the trial is not long.

For peer review only

Introduction

For peer review only - http://bmjopen.bmj.com/site/about/guidelines.xhtml

Globally around 15 million people suffer from stroke each year and a quarter of them experience permanent disability[1]. Much of this burden is borne by Low and Middle Income Countries (LMICs) [2]. The increase in prevalence of stroke[3] and consequently of stroke-related disability may cause an overwhelming demand for rehabilitation services worldwide[3]. This situation is especially of concern for LMICs like India where the resources for rehabilitation are often limited[3].

Stroke is one of the leading causes of death and disability in India. Given the paucity of data on stroke in India, a systematic review of population-based studies on stroke in India was conducted. Studies included in this review showed that the crude stroke prevalence during the past two decades in India ranged from 44/100,000 persons to 559/100,000 persons, [4] and the cumulative incidence of stroke in India ranged from 105-152/100,000 person per year[4]. These estimates on stroke incidence and prevalence are found to be higher than those reported from High Income Countries[5.] The growing burden of stroke-related disability and the unmet need for rehabilitation following stroke in India poses a major public health challenge.

There is a paucity of global evidence on the effectiveness of therapy-based stroke rehabilitation, especially in long-term care [6-7]. Available evidence shows that there is no single physical rehabilitation approach that is more effective than combinations of care[8]. Provision of information to stroke survivors and caregivers has been shown to

For peer review only - http://bmjopen.bmj.com/site/about/guidelines.xhtml

improve functional outcomes[9]. However, the best way to do this is still unclear. Recently, mHealth options are rising substantially and mobile technology has been substantially used to communicate for health-related reasons. Though mHealth strategies have developed various solutions to meet the needs of stroke survivors, the best way to utilise this approach in stroke rehabilitation is also still unclear[10]. There is insufficient evidence for tele-rehabilitation services[11]. This context provides a strong grounding for the development of cost-effective multi-dimensional stroke rehabilitation interventions to meet the demands of the stroke survivors. In the absence of organised stroke care services, and with the limited resources available for rehabilitation, a comprehensive approach to address the growing burden of stroke-related disability in India becomes pertinent[12]. This approach could be pivotal in integrating various strategies for rehabilitation[3] (Educational, Community-based rehabilitation, digital technology, Self/Supported management etc.). It could also be useful for targeting the full range of impacts of stroke, including on impairments, activity limitations and participation restriction, as outlined in the 'Biopsychosocial conceptualization of disability framework' for the intervention, as proposed by the ICF[13].

A smartphone-enabled carer-supported educational intervention was developed by our group for the management of physical disabilities following stroke in India[14].

For peer review only - http://bmjopen.bmj.com/site/about/guidelines.xhtml

This intervention was named as ‘Care for Stroke’. It was developed using the systematic approach to development and evaluation of complex interventions, as recommended by the Medical Research Council (MRC) in the U.K. [15-16]. We intended to bridge the gaps in access to stroke services through this innovative intervention which optimises relevant public health practice with the support from mobile devices such as smartphones, personal digital assistants and other wireless devices[17]. To the best of our knowledge, there is no other stroke rehabilitation intervention enabled through mHealth platforms that are available and relevant to India.

The intervention was evaluated for its feasibility and acceptability in an Indian context[18]. The intervention includes information about stroke and the ways to manage physical disability following stroke. It contains a practical demonstration of functional post-stroke exercises to acquire the functional abilities necessary to perform everyday tasks, adaptive techniques to perform one’s own daily activities independently and a specific section on assistive devices that could enable participation of the stroke survivors in their daily tasks[14]. Findings from the pilot-testing showed that the ‘Care for Stroke’ intervention was feasible and acceptable in the Indian context[18]. About 95% of the stroke survivors and all the caregivers (100%) rated the intervention as “excellent”, based on it’s a) overall credibility, b) feasibility and c) user-friendliness[18].

For peer review only - http://bmjopen.bmj.com/site/about/guidelines.xhtml

However, feasibility and acceptability alone will not be sufficient to inform implementation and scalability[16]. Nor will it be enough in order to advocate for change in policy towards implementation of an intervention[19]. Investigating the intervention clinical and cost effectiveness will provide insights for planning, implementation and the potential scalability of the intervention, especially in countries with limited resources. Given the methodological quality of the available evidence [9-11], there is a pressing need to conduct a rigorous (randomized, controlled, sufficiently powered) clinical trial to demonstrate the effectiveness of the 'Care for Stroke' intervention.

Objective:

The objective of the randomised controlled trial is to evaluate whether the 'Care for Stroke' intervention is effective for the reduction of dependency in activities of daily living among stroke survivors compared to people receiving standard treatment in an India setting. The primary outcome for the study is disability measured by the Modified Rankin Scale.

Methods:

Overview

For peer review only - http://bmjopen.bmj.com/site/about/guidelines.xhtml

This trial will be a pragmatic, randomised, outcome assessor-blinded trial to quantify the effectiveness of the Care for Stroke Intervention on reducing dependency in activities of daily living following stroke. A total of 266 adult stroke survivors who fulfil the eligibility criteria will be randomised to receive either 'Care for Stroke' intervention or standard treatment and will be followed for six weeks. The flow chart of the entire trial process is provided in figure - 1.

Pragmatic design and the uncertainty principle

The effectiveness of the intervention in routine practice can be assessed using the pragmatic trial design. Until now, there is no evidence for effectiveness of stroke rehabilitation interventions that is unidisciplinary, led by a physician, neurologist or a physiotherapist alone[12]. However, a physiotherapist or physician-driven unidisciplinary rehabilitation is what is commonly practiced in the context of stroke rehabilitation in India[12]. Given the lack of evidence, there is a natural uncertainty among the health professionals involved in provision of stroke care about what intervention could work best for the stroke survivors in an Indian context. The eligibility for participant recruitment in the 'Care for Stroke Intervention' trial will be based on this uncertainty principle. This approach to assess participant eligibility is well established[20].

Setting

Participant Recruitment

For peer review only - http://bmjopen.bmj.com/site/about/guidelines.xhtml

Participants will be identified using their contact details from treatment records for their first ever stroke. These details for stroke survivors in India exist in two places. Participant diagnosis and details can be collected from the hospital records from which an individual received treatment for his/her stroke. It is also available at the government health insurance department where the cost of the treatment for stroke is covered by this insurance department. Hence participants will be identified through both these options. The identified participants will be contacted at their home for consent and recruitment. The intervention will be provided to the participants at home and they will be asked to use the intervention in their home.

Eligible Participants:

Inclusion Criteria

- Adults (aged ≥18 years)
- Recent diagnosis of first-ever stroke as defined by the WHO [21]
- Any level of stroke severity (score 1 - 42, according to NIH stroke scale[22-23])
- Stroke survivor medically stable (reaching a point in medical treatment where life-threatening problems following stroke have been brought under control)
- Post-stroke functional status of the stroke survivor: requiring assistance of at least one person to perform daily activities such as transfers, self-care and mobility (i.e. scoring less than the maximum score obtainable in one or more components of the Barthel Index[24])

For peer review only
For peer review only - http://bmjopen.bmj.com/site/about/guidelines.xhtml

- Stroke survivor residing with a primary caregiver (family member) at home.

Exclusion Criteria

- Severe cognitive difficulties (scoring >1 in Orientation, Executive function, Inattention and Language components of the NIH Stroke Scale for cognition [25])
- Severe communication problem (scoring >1 in Dysarthria and Best Language component of the NIH Stroke Scale [22-23])
- Stroke survivor functionally dependent because of other pre-existing conditions (e.g. amputation, fracture, dementia)
- Stroke survivor without a primary caregiver
- Stroke survivor unwilling/unable to adhere to the study protocol
- Stroke survivors who did not meet the training requirements regarding operation of a smartphone. This criterion was deliberately placed just to make sure that there is no dropout after the recruitment. It was based on the observations from previous piloting.

Randomisation

For peer review only - http://bmjopen.bmj.com/site/about/guidelines.xhtml

Stroke survivors will receive all-usual treatment for stroke. Participants eligible for inclusion will be identified by a trial investigator. The eligible participants will be initially contacted by telephone and they will be visited in person at their home by the investigator to share the details about the study to the participant and the identified caregiver. A participant information sheet outlining the study objectives, risks and benefits along with brief information sheet about stroke will be provided to the participant. Written informed consent for participation in the intervention will be sought from all participants or from the next of kin if the participant is unable to consent.

An entry form will be used to collect baseline information including the contact details of the participant and the identified caregiver. This information will be forwarded to the independent randomisation centre and the participants eligible for inclusion will be randomised to the intervention or control arm in a 1:1 ratio using a secure, central, password-protected, web-based system. The intervention will be started within 24 hours of randomisation.

Sample size estimation

The two main factors that determine the number of participants needed in this trial are the estimated event rate and the size of the treatment effect. The primary

For peer review only - http://bmjopen.bmj.com/site/about/guidelines.xhtml

outcome for the 'Care for Stroke' trial is dependency in activities of daily living measured at six weeks post recruitment.

Estimated event rate: In a meta-analysis of early supported discharge trial among participants with stroke, 50% of the stroke survivors were either dead or dependent at the end of follow-up and the beneficial effect of the intervention in the treatment group was an odds reduction of 21% of death and dependency[26].

As a non-inferiority one-sided trial, to evaluate the effectiveness of the Smartphone-enabled educational intervention on dependency, there will be a requirement of 266 participants (133 in each group) to detect a 20% difference in dependency among the participants between the treatment groups with 90% power at the 5 % level of statistical significance and with 20% loss to follow up.

A non-inferiority trial could exclude the possibility of a small degree of inferiority of a new intervention relative to an active control given the sample size. The results of the trial provided by the confidence interval will allow concrete evaluation of the precision actually achieved, superseding any power calculation carried out before the starting the trial.

For peer review only - http://bmjopen.bmj.com/site/about/guidelines.xhtml

Intervention

The 'Care for Stroke' intervention will be delivered through a smartphone and it will include information about stroke and the ways to manage post-stroke disabilities. The intervention includes 2-3 minutes of 60 videos in vernacular language organized in five sections. The sections are: 1) information about stroke, 2) home-based exercises, 3) functional skills training, 4) activities of daily living, and 5) assistive devices. The intervention will be self-directed, with participants seeking information in the different categories as they require. The intervention will also have an option for the stroke survivor or the identified caregiver to contact the intervention provider for any technical support in accessing the intervention through Smartphone.

Intervention Arm

The stroke survivor and their caregiver will receive 45–60 min of training on accessing and use of the intervention (watching videos) via the smartphone. Participants will then be provided with a smartphone preloaded with the 'Care for Stroke' intervention and asked to try it out on their own. Three or more errorless attempts to retrieve any required part of the intervention from the smartphone will be considered successful training. After successful training, participants will be provided with a smartphone loaded with the intervention and will be asked to use this intervention at their discretion at home over a six week period.

For peer review only - http://bmjopen.bmj.com/site/about/guidelines.xhtml

The identified caregivers of stroke survivors will be asked to support the stroke survivors as and when necessary to access the intervention from the smartphone. The participants will be telephonically supported at least once in a week during the intervention period. The telephonic support is essentially to remind and obtain updates from the participants or identified caregivers on utilisation of the intervention. A summary of this conversation will be documented and the notes will be kept privately in a locked cupboard. The participants in the intervention arm will not be restricted from receiving standard treatment for their stroke.

Control Arm

Participants in the control arm will receive standard post stroke rehabilitation services. In general, the standard treatment may include provision of physiotherapy (45 minutes to 60 minutes) at home or in a clinic facility for the stroke survivors based on goals set by the specific therapist or a rehabilitation team.

Outcome Measures

Primary Outcome

The primary outcome measure is dependency in activities of daily living and will be measured by the Modified Rankin Scale [27] (MRS) at baseline and at six weeks after randomisation. The MRS scale measures the degree of disability or dependence in the activities of daily living of people who have suffered a stroke in six categories.

For peer review only - http://bmjopen.bmj.com/site/about/guidelines.xhtml

The scores range from zero (no symptoms) to a maximum of six (dead). A dichotomous approach to outcome analysis will be used. Participants' scores will be categorised into MRS scores of 0-3 and 4-6.

Secondary Outcome

Secondary outcome measures will be:

- Modified Barthel Index [24]
- Modified Caregiver Strain Index [28]
- Quality of Life measured by WHOQOL – BREF [29]
- Use of Health care and Rehabilitation services (Therapy, Hospitalisation and medication, AYUSH, traditional practices etc.)

This information will be collected through questionnaire at baseline and after 6 weeks. The Smartphone application has an inbuilt monitoring mechanism where the usage of the intervention by the participants will be tracked.

Costs for rehabilitative care will be collected from participants both in the treatment groups to see whether the Care for Stroke intervention delivered through a smartphone reduces the overall costs of care (cost-effectiveness).

- Direct costs of health care and rehabilitation since the time of stroke
- Indirect costs (A family member giving up paid employment and taking the role of a caregiver, travel costs etc.)

For peer review only

For peer review only - http://bmjopen.bmj.com/site/about/guidelines.xhtml

Follow up

An outcome form will be completed at six weeks after randomisation or at death, if either happens sooner. A blinded outcome assessor will evaluate all the outcomes (primary and secondary) at baseline and at six weeks. A relatively short follow-up period has been selected as The Stroke Therapy Academic Industry Round Table (STAIRS) strongly recommends a shorter follow-up period to reduce variation in clinical outcome that could occur due to subsequent stroke events that are unrelated to the trial[24]. This will also allow accurate assessment of the outcome [30].

Adverse events

Adverse events are very common among acute stroke survivors. Some of the expected adverse events during the trial are

1. Death due to any vascular causes (e.g. myocardial infarction, recurrent stroke),
2. Hospitalization due to post-stroke complications such as infections, brain oedema, seizures, deep vein thrombosis, urinary tract infections, pressure sores and shoulder subluxation, dislocation and fracture.
3. Occurrence of secondary stroke.

These events will be documented during follow-up telephone calls and it will be presented to an independent data safety and monitoring committee for unblinded review.

For peer review only - http://bmjopen.bmj.com/site/about/guidelines.xhtml

Data Collection and Management

This trial will be centrally coordinated from the trial coordination center (TCC) at the Indian Institute of Public Health (IIPH) Hyderabad. Baseline data will be collected by the investigator and follow-up data will be collected with appropriate translation by an independent blinded outcome assessor on paper forms. These data will be securely scanned and sent to the TCC for entry into the password protected secured electronic database. An independent data safety and monitoring committee (DSMC) will be set up to monitor data collection and management. A trial steering committee will also be set up to oversee the conduct of the trial.

Analysis

The main analyses will compare all those allocated to the 'Care for Stroke' intervention versus those allocated to the standard treatment group on an 'intention-to-treat' basis, irrespective of whether the participants received the treatment allocated or not. The imbalance in recruiting equal number of participants if any will be addressed during the analysis phase using appropriate statistical techniques. The dichotomized MRS scores (0-3 and 4-6) in both the groups will be used to calculate the effect estimates with a measure of precision (95% confidence interval) and presented in the results of the trial. Subgroup analysis for the primary outcome will be based on stroke severity, location of the participant (urban/rural), gender and age at stroke. Interaction tests will also be used to test whether the effect of treatment (if any) differs across these subgroups.

For peer review only - http://bmjopen.bmj.com/site/about/guidelines.xhtml

Recruitment of participants:

The trial will identify and recruit participants from the hospital records as well as stroke insurance records available at the Aarogyasri trust until the sample size is achieved. Currently, the average stroke insurance claim rate through this trust is 10-12 stroke survivors per month. Hence it would take approximately 32-36 months for recruiting the proposed number of participants in this trial.

Patient and Public Involvement:

Patients and public were not involved for the purpose of protocol development.

Ethics and Dissemination

Ethical approval for this trial has been obtained from the independent institutional research ethics committee at the public health foundation of India (IIPH) Hyderabad. Results of this trial will be published in relevant, peer-reviewed, indexed, international journal.

For peer review only

For peer review only - http://bmjopen.bmj.com/site/about/guidelines.xhtml

References

1. Global Burden of Disease Collaborative Network. Global Burden of Disease Study 2016 (GBD 2016) Disability-Adjusted Life Years and Healthy Life Expectancy 1990-2016. Seattle, United States: Institute for Health Metrics and Evaluation (IHME), 2017.
2. Ferri CP, Schoenborn C, Kaira L, Acosta D, Guerra M, Huang Y, Jacob KS, Llibre Rodriquez JJ, Salas A, Sosa AL, et al. Prevalence of stroke and related burden among older people living in Latin America, India and China. J Neurol Neurosurg Psychiatry 2011;82:1074
3. World report on disability. Geneva, Switzerland: WHO, 2011. http://www.who.int/disabilities/world_report/2011/report/en/
4. Kamalakannan Sureshkumar, Gudlavalleti Aashrai S. V., Gudlavalleti Venkata S. Murthy, Goenka Shifalika, Kuper Hannah. Incidence & prevalence of stroke in India: A systematic review. 2017; 146 (2): 175-185
5. Feigin VL, Lawes CM, Bennett DA, Barker-Collo SL, Parag V. Worldwide stroke incidence and early case fatality reported in 56 population-based studies: a systematic review. *Lancet Neurology*. 2009; 8 (4): 355-369.

For peer review only - http://bmjopen.bmj.com/site/about/guidelines.xhtml

6. OutParticipant Service Trialists. Therapy-based rehabilitation services for stroke Participants at home. *Cochrane Database of Systematic Reviews.* 2003, Issue 1. Art. No.: CD002925. DOI: 10.1002/14651858.CD002925.
7. Turner-Stokes L, Nair A, Sedki I, Disler PB, Wade DT. Multi-disciplinary rehabilitation for acquired brain injury in adults of working age. *Cochrane Database of Systematic Reviews.* 2005, Issue 3. Art. No.: CD004170. DOI: 10.1002/14651858.CD004170.pub2.
8. Forster A, Brown L, Smith J, House A, Knapp P, Wright JJ, Young J. Information provision for stroke Participants and their caregivers. *Cochrane Database of Systematic Reviews.* 2012, Issue 11. Art. No.: CD001919. DOI: 10.1002/14651858.CD001919.pub3.
9. Sarfo FS, Ovbiagele B. Mobile health for stroke: a promising concept for research and practice. *mHealth*. 2017; 3: 4. doi:10.21037/mhealth.2017.02.01.
10. Laver KE, Schoene D, Crotty M, George S, Lannin NA, Sherrington C. Telerehabilitation services for stroke. *Cochrane Database of Systematic Reviews.* 2013, Issue 12. Art. No.: CD010255. DOI: 10.1002/14651858.CD010255.pub2.
11. Laver KE, George S, Thomas S, Deutsch JE, Crotty M. Virtual reality for stroke rehabilitation. *Cochrane Database of Systematic Reviews.* 2015, Issue 2. Art. No.: CD008349. DOI: 10.1002/14651858.CD008349.pub3.
12. Kamalakannan, Sureshkumar et al. Rehabilitation needs of Stroke Survivors after Discharge from Hospital in India. Archives of Physical Medicine and

For peer review only - http://bmjopen.bmj.com/site/about/guidelines.xhtml

Rehabilitation; 2016. Volume 97, Issue 9, 1526 - 1532.e9.

13. WHO. *The International Classification of Functioning, Disability and Health*. World Health Organization. Geneva, Switzerland: WHO, 2001.
14. Sureshkumar K, Murthy GVS, Munuswamy S, S. Goenka and H Kuper. 'Care for Stroke' a web-based, Smartphone-enabled educational intervention for management of physical disabilities following stroke: Feasibility in the Indian context. *BMJ Innovations* 2015; 1 127–36.
15. Peter Craig, Mark Petticrew, Developing and evaluating complex interventions: Reflections on the 2008 MRC guidance. *International Journal of Nursing Studies.* 2013; 50 (5): 585-87.
16. Craig P. Foreword. In: Richards D, Rahm Hallberg I. *Complex interventions in health: an overview of research methods.* Routledge, 2015.
17. World Health Organization mHealth: New Horizons for Health through Mobile Technologies: Based on the Findings of the Second Global Survey on eHealth (Global Observatory for eHealth Series, Volume 3) 2011. [2013-0523].
18. Sureshkumar K, Murthy GVS, Natarajan S, Naveen C, Goenka S, Kuper H. Evaluation of the feasibility and acceptability of the 'Care for Stroke' intervention in India, a smartphone-enabled, carer-supported, educational intervention for management of disability following stroke. BMJ Open 2016;6:e009243. doi:10.1136/bmjopen-2015009243

For peer review only - http://bmjopen.bmj.com/site/about/guidelines.xhtml

19. Bowen S, Zwi AB. Pathways to "Evidence-Informed" Policy and Practice: A Framework for Action. *PLoS Medicine*. 2005;2(7):e166. doi:10.1371/journal.pmed.0020166.

20. Peto R, Baigent C. Trials: the next 50 years : Large scale randomised evidence of moderate benefits . *BMJ : British Medical Journal*. 1998;317(7167):1170-1171.

21. WHO MONICA Project Investigators. The World Health Organization MONICA Project (Monitoring trends and determinants in cardiovascular disease). *J Clin Epidemiol*. 1988; (41): 105-114.

22. Department of Health and Human Services. The National Institute of Neurological Disorders and Stroke (NINDS). *NIH Stroke Scale Training, Part 2. Basic Instruction*. 2010. https://archive.org/details/gov.hhs.ninds.stroke.1.2 (accessed 3 August 2010).

23. Ver Hage A. The NIH stroke scale: a window into neurological status. Nurse.Com; *Nursing Spectrum* (Greater Chicago). 2011; 24 (15):44-49.

24. Mahoney F. Barthel D. Functional evaluation: the Barthel Index. *Md Med J*.1965; 14:61–65.

25. Cumming TB, Blomstrand C, Bernhardt J, Linden T. The NIH stroke scale can establish cognitive function after stroke. *Cerebrovasc Dis*. 2010; 30(1):7-14.

26. Langhorne P, Taylor G, Murray G. Dennise M, Anderson C, Bautz Holter E, Dey P, Indredavik B, Mayo N, Power M, et al. Early supported discharge

For peer review only - http://bmjopen.bmj.com/site/about/guidelines.xhtml

services for stroke Participants: a meta-analysis of individual Participants' data. Lancet. 2005; 365(9458):501–6.

27. Rankin L. Cerebral vascular accidents in Participants over the age of 60. II. Prognosis. *Scott Med J.* 1957; 2: 200-215.
28. Thornton, M., & Travis, S.S. Analysis of the reliability of the Modified Caregiver Strain Index. The Journal of Gerontology, Series B, 2003. Psychological Sciences and Social Sciences, 58B(2), S127-132.
29. World Health Organization's. Quality of Life group: WHOQOL-sBREF Introduction. Administration and Scoring. Field Trial version. 1996
30. Aziz NA, Leonardi-Bee J, Phillips MF, Gladman JR, Legg L, Walker MF. Therapy-based rehabilitation services for Participants living at home more than one year after stroke. *Cochrane Database of Systematic Reviews.* 2008, Issue 2. Art. No.: CD005952. DOI: 10.1002/14651858.CD005952.pub2.

Authors' Contributions

K Sureshkumar (SK) conceived, designed and drafted the manuscript. Prof GVS Murthy and Dr Hannah Kuper played a crucial role in conception of the research study and provided substantial guidance in designing and conducting evaluation.

Funding

For peer review only - http://bmjopen.bmj.com/site/about/guidelines.xhtml

This work was funded and supported **The Wellcome Trust DBT India Alliance.** Grant Code: IA/CPHE/16/1/502650

Competing Interests

The authors declare that they have no competing interests, financial or non-financial.

Acknowledgement

We thank The Wellcome-trust-DBT India Alliance for funding the research study. We thank the independent institutional research ethics committee of the PHFI-Indian Institute of Public Health - Hyderabad for granting scientific and ethics approval to conduct this research study. We thank the consultants from Suchir softech and Selva photography for developing the software application and digitization of the content of the intervention.

Figure Legends

1. Figure – 1: Flow Chart of the Care for Stroke Trial

For peer review only - http://bmjopen.bmj.com/site/about/guidelines.xhtml

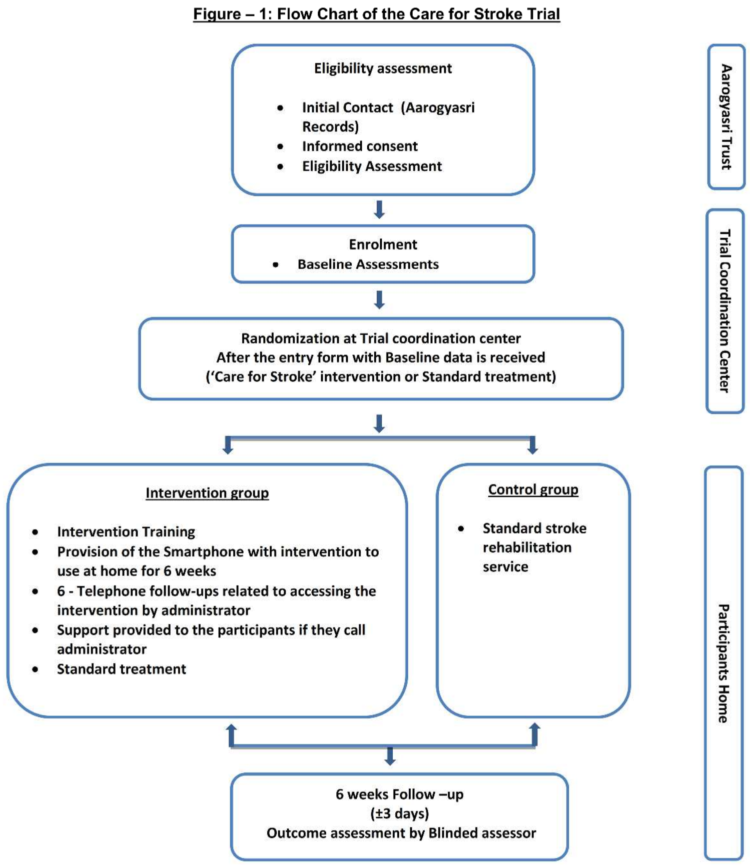

Figure – 1: Flow Chart of the Care for Stroke Trial Process

SPIRIT
STANDARD PROTOCOL ITEMS: RECOMMENDATIONS FOR INTERVENTIONAL TRIALS

SPIRIT 2013 Checklist: Recommended items to address in a clinical trial protocol and related documents*

Section/item	Item No	Description	Addressed on page number
**Administrative information**			
Title	1	Descriptive title identifying the study design, population, interventions, and, if applicable, trial acronym	1
Trial registration	2a	Trial identifier and registry name. If not yet registered, name of intended registry	4
	2b	All items from the World Health Organization Trial Registration Data Set	4
Protocol version	3	Date and version identifier	1
Funding	4	Sources and types of financial, material, and other support	25
Roles and responsibilities	5a	Names, affiliations, and roles of protocol contributors	1
	5b	Name and contact information for the trial sponsor	25
	5c	Role of study sponsor and funders, if any, in study design; collection, management, analysis, and interpretation of data; writing of the report; and the decision to submit the report for publication, including whether they will have ultimate authority over any of these activities	N/A
	5d	Composition, roles, and responsibilities of the coordinating centre, steering committee, endpoint adjudication committee, data management team, and other individuals or groups overseeing the trial, if applicable (see Item 21a for data monitoring committee)	19

For peer review only

For peer review only - http://bmjopen.bmj.com/site/about/guidelines.xhtml

Introduction

Background and rationale	6a	Description of research question and justification for undertaking the trial, including summary of relevant studies (published and unpublished) examining benefits and harms for each intervention	6-8
	6b	Explanation for choice of comparators	6-8
Objectives	7	Specific objectives or hypotheses	9
Trial design	8	Description of trial design including type of trial (eg, parallel group, crossover, factorial, single group), allocation ratio, and framework (eg, superiority, equivalence, noninferiority, exploratory)	9,14

Methods: Participants, interventions, and outcomes

Study setting	9	Description of study settings (eg, community clinic, academic hospital) and list of countries where data will be collected. Reference to where list of study sites can be obtained	11
Eligibility criteria	10	Inclusion and exclusion criteria for participants. If applicable, eligibility criteria for study centres and individuals who will perform the interventions (eg, surgeons, psychotherapists)	11
Interventions	11a	Interventions for each group with sufficient detail to allow replication, including how and when they will be administered	14-15
	11b	Criteria for discontinuing or modifying allocated interventions for a given trial participant (eg, drug dose change in response to harms, participant request, or improving/worsening disease)	17
	11c	Strategies to improve adherence to intervention protocols, and any procedures for monitoring adherence (eg, drug tablet return, laboratory tests)	14-15
	11d	Relevant concomitant care and interventions that are permitted or prohibited during the trial	14-15
Outcomes	12	Primary, secondary, and other outcomes, including the specific measurement variable (eg, systolic blood pressure), analysis metric (eg, change from baseline, final value, time to event), method of aggregation (eg, median, proportion), and time point for each outcome. Explanation of the clinical relevance of chosen efficacy and harm outcomes is strongly recommended	16-17
Participant timeline	13	Time schedule of enrolment, interventions (including any run-ins and washouts), assessments, and visits for participants. A schematic diagram is highly recommended (see Figure)	Figure-1

For peer review only - http://bmjopen.bmj.com/site/about/guidelines.xhtml

Sample size	14	Estimated number of participants needed to achieve study objectives and how it was determined, including clinical and statistical assumptions supporting any sample size calculations	13
Recruitment	15	Strategies for achieving adequate participant enrolment to reach target sample size	11

Methods: Assignment of interventions (for controlled trials)

Allocation:

Sequence generation	16a	Method of generating the allocation sequence (eg, computer-generated random numbers), and list of any factors for stratification. To reduce predictability of a random sequence, details of any planned restriction (eg, blocking) should be provided in a separate document that is unavailable to those who enrol participants or assign interventions	12-13
Allocation concealment mechanism	16b	Mechanism of implementing the allocation sequence (eg, central telephone; sequentially numbered, opaque, sealed envelopes), describing any steps to conceal the sequence until interventions are assigned	12-13
Implementation	16c	Who will generate the allocation sequence, who will enrol participants, and who will assign participants to interventions	12-13
Blinding (masking)	17a	Who will be blinded after assignment to interventions (eg, trial participants, care providers, outcome assessors, data analysts), and how	17
	17b	If blinded, circumstances under which unblinding is permissible, and procedure for revealing a participant's allocated intervention during the trial	18

Methods: Data collection, management, and analysis

Data collection methods	18a	Plans for assessment and collection of outcome, baseline, and other trial data, including any related processes to promote data quality (eg, duplicate measurements, training of assessors) and a description of study instruments (eg, questionnaires, laboratory tests) along with their reliability and validity, if known. Reference to where data collection forms can be found, if not in the protocol	17-18
	18b	Plans to promote participant retention and complete follow-up, including list of any outcome data to be collected for participants who discontinue or deviate from intervention protocols	16-17

Data management	19	Plans for data entry, coding, security, and storage, including any related processes to promote data quality (eg, double data entry; range checks for data values). Reference to where details of data management procedures can be found, if not in the protocol	19
Statistical methods	20a	Statistical methods for analysing primary and secondary outcomes. Reference to where other details of the statistical analysis plan can be found, if not in the protocol	19
	20b	Methods for any additional analyses (eg, subgroup and adjusted analyses)	19
	20c	Definition of analysis population relating to protocol non-adherence (eg, as randomised analysis), and any statistical methods to handle missing data (eg, multiple imputation)	19
**Methods: Monitoring**			
Data monitoring	21a	Composition of data monitoring committee (DMC); summary of its role and reporting structure; statement of whether it is independent from the sponsor and competing interests; and reference to where further details about its charter can be found, if not in the protocol. Alternatively, an explanation of why a DMC is not needed	19
	21b	Description of any interim analyses and stopping guidelines, including who will have access to these interim results and make the final decision to terminate the trial	19
Harms	22	Plans for collecting, assessing, reporting, and managing solicited and spontaneously reported adverse events and other unintended effects of trial interventions or trial conduct	19
Auditing	23	Frequency and procedures for auditing trial conduct, if any, and whether the process will be independent from investigators and the sponsor	19
**Ethics and dissemination**			
Research ethics approval	24	Plans for seeking research ethics committee/institutional review board (REC/IRB) approval	20
Protocol amendments	25	Plans for communicating important protocol modifications (eg, changes to eligibility criteria, outcomes, analyses) to relevant parties (eg, investigators, REC/IRBs, trial participants, trial registries, journals, regulators)	20

For peer review only - http://bmjopen.bmj.com/site/about/guidelines.xhtml

Consent or assent	26a	Who will obtain informed consent or assent from potential trial participants or authorised surrogates, and how (see Item 32)	13
	26b	Additional consent provisions for collection and use of participant data and biological specimens in ancillary studies, if applicable	13
Confidentiality	27	How personal information about potential and enrolled participants will be collected, shared, and maintained in order to protect confidentiality before, during, and after the trial	18
Declaration of interests	28	Financial and other competing interests for principal investigators for the overall trial and each study site	25
Access to data	29	Statement of who will have access to the final trial dataset, and disclosure of contractual agreements that limit such access for investigators	18-19
Ancillary and post-trial care	30	Provisions, if any, for ancillary and post-trial care, and for compensation to those who suffer harm from trial participation	N/A
Dissemination policy	31a	Plans for investigators and sponsor to communicate trial results to participants, healthcare professionals, the public, and other relevant groups (eg, via publication, reporting in results databases, or other data sharing arrangements), including any publication restrictions	20
	31b	Authorship eligibility guidelines and any intended use of professional writers	N/A
	31c	Plans, if any, for granting public access to the full protocol, participant-level dataset, and statistical code	4,20
**Appendices**			
Informed consent materials	32	Model consent form and other related documentation given to participants and authorised surrogates	NO
Biological specimens	33	Plans for collection, laboratory evaluation, and storage of biological specimens for genetic or molecular analysis in the current trial and for future use in ancillary studies, if applicable	N/A

*It is strongly recommended that this checklist be read in conjunction with the SPIRIT 2013 Explanation & Elaboration for important clarification on the items. Amendments to the protocol should be tracked and dated. The SPIRIT checklist is copyrighted by the SPIRIT Group under the Creative Commons "Attribution-NonCommercial-NoDerivs 3.0 Unported" license.
